# Microbial hydrogen “manufactory” for enhanced gas therapy and self-activated immunotherapy via reduced immune escape

**DOI:** 10.1186/s12951-022-01440-7

**Published:** 2022-06-15

**Authors:** Hongyu Yan, Miao Fan, Huifang Liu, Tingshan Xiao, Dandan Han, Ruijun Che, Wei Zhang, Xiaohan Zhou, June Wang, Chi Zhang, Xinjian Yang, Jinchao Zhang, Zhenhua Li

**Affiliations:** 1 Dongguan Institute of Clinical Cancer Research, Affiliated Dongguan Hospital, Southern Medical University, Dongguan, 523059 China; 2 College of Chemistry & Environmental Science, Key Laboratory of Medicinal Chemistry and Molecular Diagnosis of Ministry of Education, Chemical Biology Key Laboratory of Hebei Province, Hebei University, Baoding, 071002 People’s Republic of China; 3 College of Pharmaceutical Science, Key Laboratory of Pharmaceutical Quality Control of Hebei Province, Hebei University, Baoding, 071002 People’s Republic of China; 4 Department of Orthopedics, Shanghai Jiao Tong University Affiliated Sixth Peoples’ Hospital, 600 Yishan Road, Shanghai, 200233 China

**Keywords:** Photosynthetic bacteria, Hydrogen therapy, Oxidative stress, Immune escaped, Immunotherapy

## Abstract

**Background:**

As an antioxidant, hydrogen (H_2_) can selectively react with the highly toxic hydroxyl radical (·OH) in tumor cells to break the balance of reactive oxygen species (ROS) and cause oxidative stress. However, due to the high diffusibility and storage difficulty of hydrogen, it is impossible to achieve long-term release at the tumor site, which highly limited their therapeutic effect.

**Results:**

Photosynthetic bacteria (PSB) release a large amount of hydrogen to break the balance of oxidative stress. In addition, as a nontoxic bacterium, PSB could stimulate the immune response and increase the infiltration of CD4+ and CD8+ T cells. More interestingly, we found that hydrogen therapy induced by our live PSB did not lead to the up-regulation of PD-L1 after stimulating the immune response, which could avoid the tumor immune escape.

**Conclusion:**

Hydrogen-immunotherapy significantly kills tumor cells. We believe that our live microbial hydrogen production system provides a new strategy for cancer hydrogen treatment combining with enhanced immunotherapy without up-regulating PD-L1.

**Graphical Abstract:**

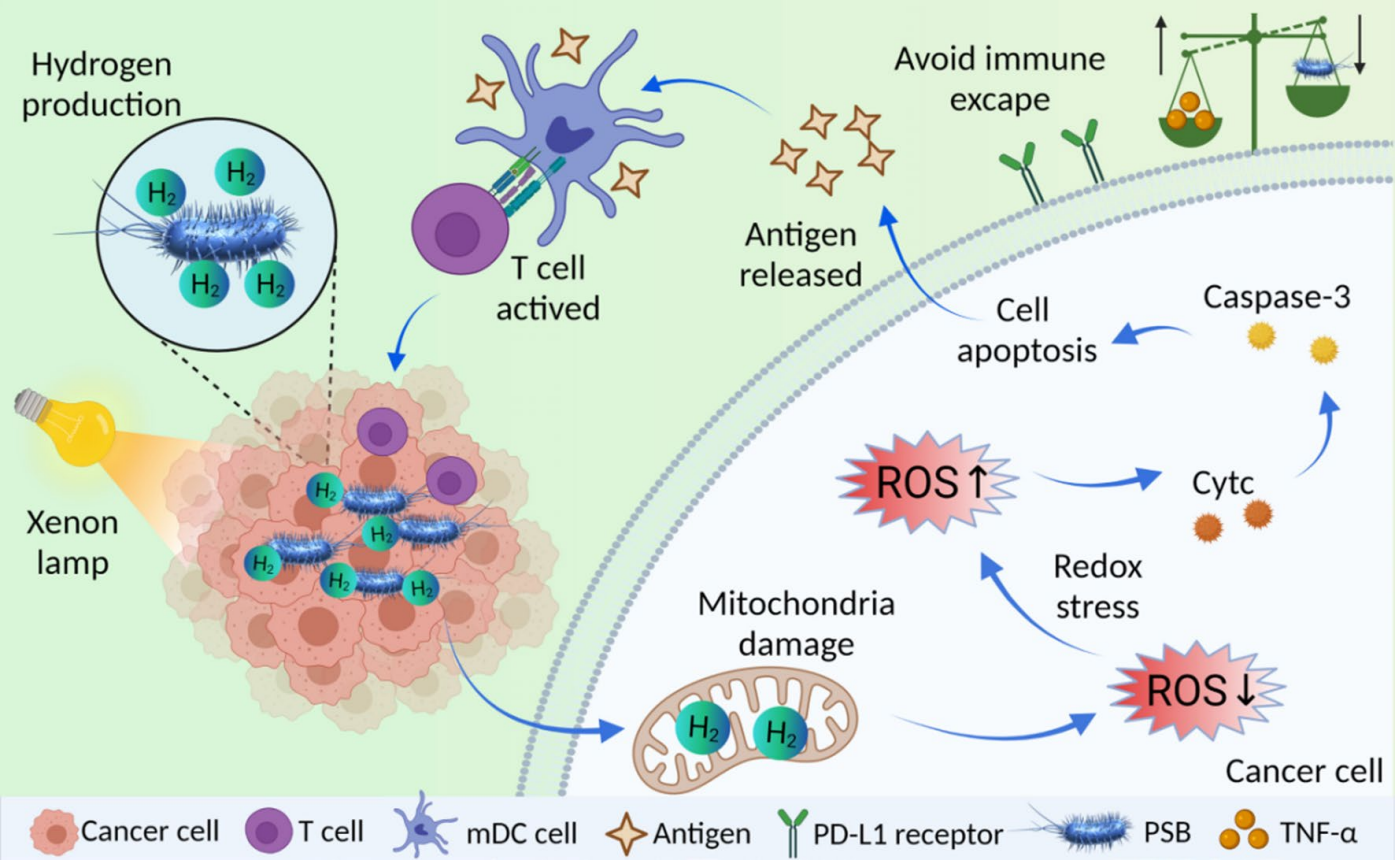

**Supplementary Information:**

The online version contains supplementary material available at 10.1186/s12951-022-01440-7.

## Introduction

As the lightest gas in nature, the biological effects of H_2_ have attracted widespread attention. As early as 1975, Dole et al. found that H_2_ could inhibit tumor growth in mice with squamous cell carcinoma [1]. H_2_ possesses the properties of biological reduction and has been found to have a good therapeutic effect in some oxidative stress and inflammatory diseases [2–11]. ROS in tumor cells were in a high balance state. H_2_ can react with highly toxic free radicals in ROS such as ·OH [12], which disrupted the steady-state balance of ROS in cancer cells and caused redox stress [13, 14]. In addition, different from chemical drugs, H_2_ is safe and non-toxic [15, 16]. Therefore, hydrogen therapy has attracted more and more attention in recent years. He et al. synthesized a series of metal palladium compounds to store and produce hydrogen for tumor H_2_ therapy [17–19]. In addition, other strategies that using tumor acidic microenvironment such as Mg, Fe nanoparticle as reductant for hydrogen production have also been fabricated [20, 21]. However, these strategies were limited by the non-biocompatible materials, and it was difficult to achieve long-term release of H_2_. Therefore, developing a new long-term hydrogen-production strategy plays a key role in improving the effect of hydrogen therapy.

Recently, the use of bacteria and other microorganisms as living carriers and therapeutic drugs for disease treatment has received widespread attention [22–24]. For example, *S. typhimurium* [25–27] and *Clostridium* [28, 29] have been widely used in tumor therapy. However, the mentioned bacteria are usually toxic and need complicated detoxication procedure. In addition, transgenic technology should be used to construct anti-tumor agents releasing engineered bacteria [30, 31]. PSB is a facultative anaerobic bacterium that could multiply in light and hypoxic environments [32, 33]. Our previous work has utilized nontoxic PSB as a hypoxia-targeting photothermal agent for tumor therapy [34]. More interestingly, we have found H_2_ was released as a product during photosynthesis of PSB [35, 36]. Chlorophyll and carotenoids of PSB captured photon and transmitted them to photosynthetic system upon light irradiation. Hydrogen was produced by the action of hydrogenase and nitrogenase [37]. Furthermore, our previous work has demonstrated PSB as a natural bacterium can increase the infiltration of CD4+ and CD8+ T lymphocytes to stimulate the immune response [34]. Thus, we believe PSB is a good hydrogen "storage container" for in situ and long-term H_2_ production in tumor hypoxia with the capacity of hypoxia and light-chemotaxis.

**Fig. 1:**
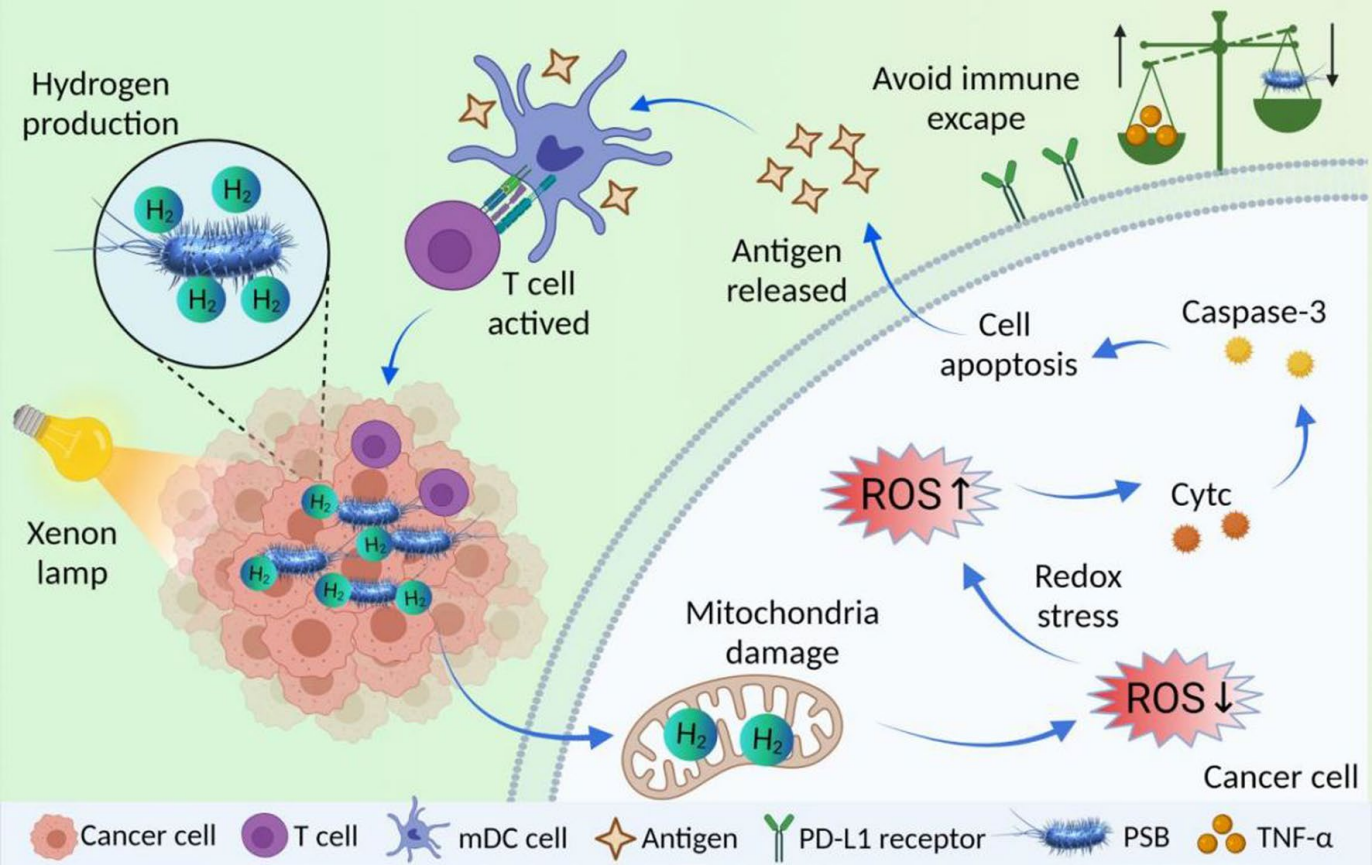
Schematic diagram. PSB with long-term hydrogen releasing capability was developed for hydrogen therapy and enhanced immunotherapy

Inspired by this, in this work, PSB with long-term hydrogen releasing capability was developed for cancer treatment. PSB autonomously targeted to the hypoxic area and continuously released hydrogen under the irradiation of xenon lamp. H_2_ diffused into cells and caused oxidative stress to kill tumor cells. Antigens were released by apoptotic cells to activate dendritic cells (DCs) and stimulate immune response. More interestingly, as chemotherapy always induce the up-regulation of PD-L1 and induce immune escape [38–41], we found that PSB-based therapy did not induce the increased expression of PD-L1, which would enhance tumor immunotherapy (Fig. 1). Thus, the combination of hydrogen therapy with self-activated immunotherapy provided a new strategy for cancer treatment.

## Results

### Characterization of PSB

**Fig. 2:**
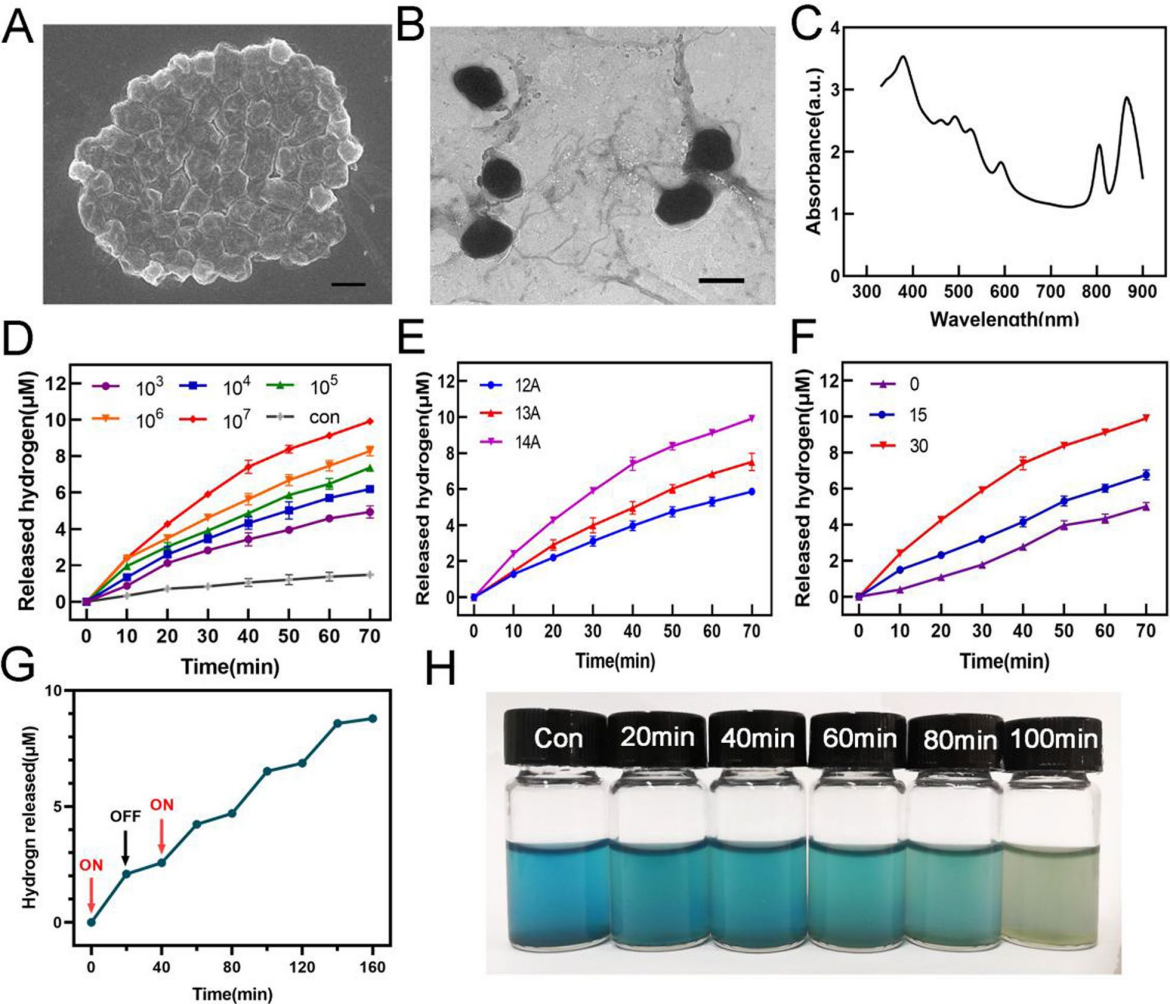
Characterization of PSB. **A** SEM image of PSB. **B** TEM image of PSB. Scale bar is 1 μm. **C** UV−Vis−NIR absorption spectra of PSB. **D** H_2_ released at different concentrations. **E** H_2_ releasing behavior produced by PSB (10^7^ CFU/mL) under various power intensities (12, 13 and 14 A) and **F** various glucose concentration (0, 15 and 30 g/L). **G** H_2_ production of PSB by irradiation with power “On” or “Off” alternately. **H** The pictures of H_2_ production in different times of radiation

The morphology of the bacterium was first characterized by scanning electron microscope (SEM) (Fig. 2A). It was found that PSB was ovoid in shape with a size of about 2 μm. Transmission electron microscope (TEM) results affirmed that PSB had flagella, which supported their motility (Fig. 2B). Gram staining showed that PSB was a gram-negative bacterium (Additional file 1: Fig. S1). As shown in the UV–Vis absorption spectrum (Fig. 2C), PSB had absorption peaks at 375 nm and 590 nm, confirming the presence of carotenoids in PSB. In addition, we found strong absorption peaks at 804 nm and 870 nm, which were characteristic absorption peaks of bacterial chlorophyll. Chlorophyll and carotenoids acted as a photon capture platform, photons were absorbed by chlorophyll and carotenoids and energy was transferred to the photosynthetic reaction center, thereby generated a high-energy electron. High-energy electrons generated ATP by cyclic phosphorylation. Nitrase and hydrogenase consumed the ATP and electrons to reduce protons to hydrogen. The hydrogen production process of PSB was completed. Then, we verified the hydrogen production performance of PSB in vitro. H_2_ was reducible and could reduce the methylene blue solution into colorless methyl blue. The absorption peak at 664 nm was measured using UV–Vis spectrum. The concentration of H_2_ production was calculated according to MB's standard curve line (Additional file 1: Fig. S2). The absorption peak showed that the amount of hydrogen production increased with the increase of PSB concentration (Fig. 2D, Additional file 1: Fig. S3) and light intensity (Fig. 2E, Additional file 1: Fig. S4). Glucose was very easy to be used by PSB. It can be used as a carbon source for PSB growth and as a hydrogen production substrate for PSB. Different glucose concentrations affected the H_2_ production of PSB. Our results indicated that hydrogen production by PSB gave a concentration-dependent behavior (Fig. 2F, Additional file 1: Fig. S5). In addition, when alternating radiation was given, it was found that H_2_ can continue production after radiation re-giving. This demonstrated the persistence of H_2_ production by PSB. Furthermore, we found that a small amount of H_2_ was still produced when switching was “OFF”. That probably because H_2_ production of PSB was a biological process that took some time to completely stop the process (Fig. 2G). Later, the production of H_2_ was shown by monitoring the color change of solution at different times. As shown in Fig. 2H, the blue color gradually grew lighter indicating the hydrogen production. Then we studied the effect of different light sources on hydrogen production using 808 laser (1 W), LED red light and xenon light (14 A) (Additional file 1: Fig. S6). Our results showed that PSB had stronger hydrogen release behavior exposure to xenon lamp comparing with other light source. Furthermore, since we have proved the PSB had a photothermal effect for cancer therapy, we studied the temperature change under the same irradiation intensity of H_2_ production. Our studies showed that the PSB induced a slight photothermal effect under xenon lamps (Additional file 1: Fig. S7) because of the low irradiation intensity. Our results indicated that H_2_ therapy may play an important role on cancer killing under low irradiation intensity.

### Hydrogen therapy effect of PSB

**Fig. 3:**
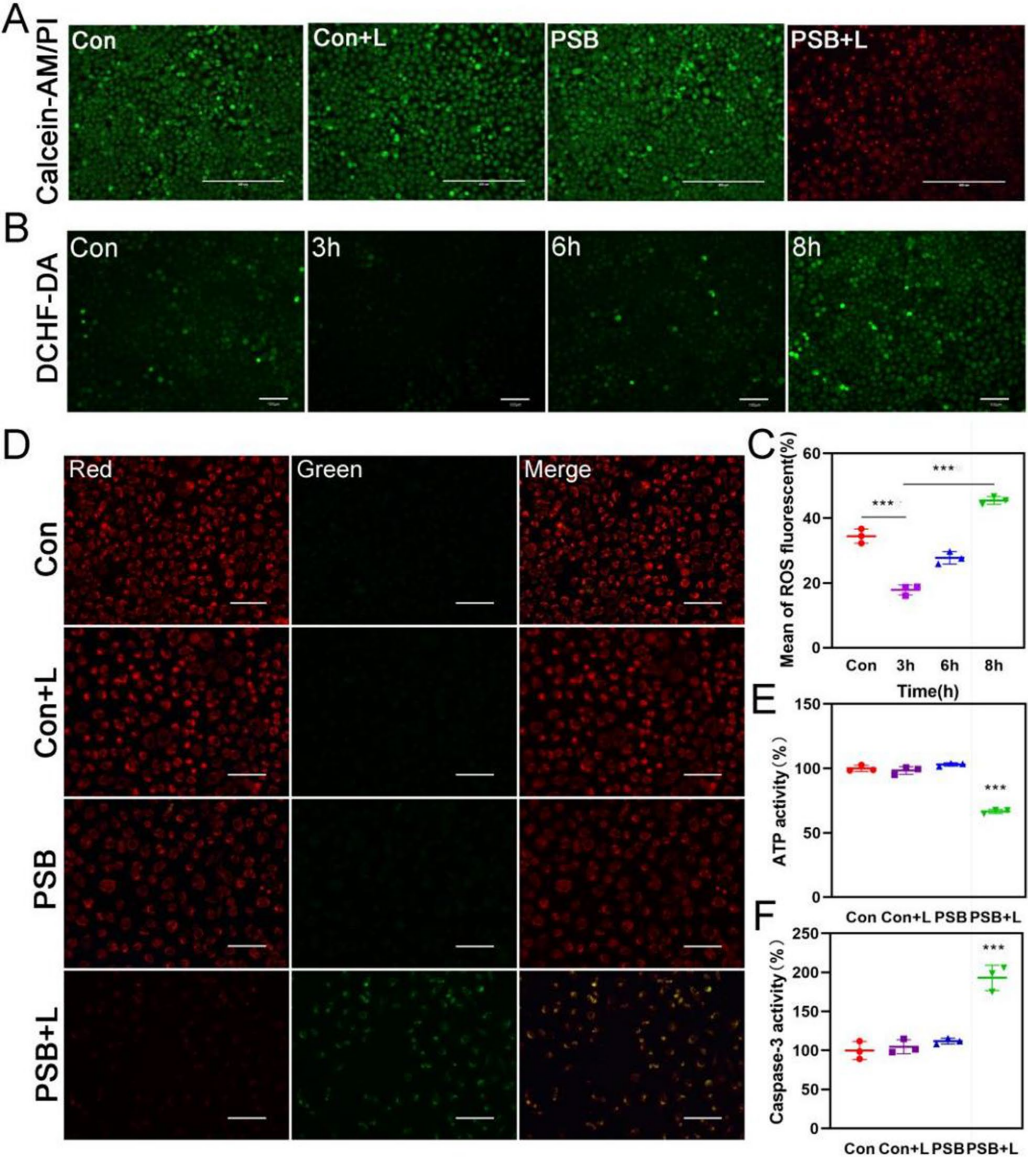
H_2_ induced apoptosis through mitochondria-dependent pathways. **A** Calcein AM and PI staining showed confocal fluorescence images of MCF-7 cells with or without xenon lamp irradiation for 10 min. (green: living cells; red: dead cells). Scale bar, 400 μm. **B** ROS changed in MCF-7 cells after hydrogen treatment. Scale bar, 100 μm. **C** Fluorescence quantification of ROS. **D** Detection of MMP changed with JC-1 staining after treatment with H_2_ for 6 h. Scale bar, 100 μm. **E** ATP activity and **F** Caspase-3 released in MCF-7 cells after treatment with H_2_. Data are presented as the mean ± s.d. (n = 3). *P < 0.05; **P < 0.01; ***P < 0.001

In order to prove the production of H_2_ and diffusion into cells, MCF-7 cells were stained with methylene blue solution and gave different treatments (Con, Con + Light, PSB, PSB + Light). As shown in Additional file 1: Fig. S8, under PSB + Light (L) treatment, the blue color was faded, which confirmed that H_2_ released by PSB and could diffuse into cells. Blue color in the other three groups had not faded. Later, MTT results showed that different concentrations of PSB had no obvious effect on cell viability, indicating their biosafety (Additional file 1: Fig. S9A). Next, live-dead cell staining analysis was used to verify the killing efficiency of hydrogen from PSB. After receiving different treatments, MCF-7 cells and MCTSs were co-stained by calcein acetoxymethyl ester and propidium iodide (Calcein AM and PI) (Fig. 3A, Additional file 1: Fig. S10). It was observed from confocal fluorescence microscope that the PSB + L group showed strong red fluorescence, while the other three groups showed green fluorescence with slight red fluorescence. It was proved that all the cells in the PSB + L group were killed and almost all the cells in the other three groups still survived. MTT results was consistent with the Live-Dead staining results indicating that H_2_ had a good killing effect on tumor cells (Additional file 1: Fig. S9B).

### H_2_ induced cells apoptosis by destroying mitochondrial function

Next, we studied the killing mechanism induced by H_2_. First, flow cytometry was used to determine the apoptosis of cells after hydrogen treatment (Additional file 1: Fig. S11). It was found that the PSB + L group induced 33.81% of cells apoptosis. The other three groups had no killing effect, indicating that H_2_ could cause cancer cells apoptosis. Tumor microenvironment contained high levels of ROS, which included a large number of highly toxic hydroxyl free radicals with a steady equilibrium. H_2_ can react with ·OH to destroy the redox balance of tumor cells by reducing the level of ROS, and then causing oxidative stress. The oxidative stress greatly increased the level of ROS. DCFH-DA fluorescence probe was used to detect the level of intracellular ROS (Fig. 3B). The fluorescence intensity in MCF-7 cells had a downward trend within 3 h, and then increased significantly. Fluorescence quantification was consistent with the previously speculated redox stress process in tumor cells (Fig. 3C). We verified the ·OH level in MCF-7 cells and found that the ·OH level was indeed up-regulated after hydrogen treatment, and the ·OH level was increase after a period of time, indicating that H_2_ caused the oxidative stress of tumor cells and increase ·OH (Additional file 1: Fig. S12). Elevated ROS levels resulted in the change of mitochondrial transmembrane potential (MMP) (Fig. 3D) and reduction of ATP (Fig. 3E). The change of MMP promoted the release of caspase-3 (Fig. 3F), which indicated that cells apoptosis was caused by the destruction of mitochondrial function by H_2_. Then we examined the mechanism of H_2_ therapy on B16-F10 cells. In consistent with H_2_ therapeutic effect in MCF-7 cells, we found H_2_ caused oxidative stress by disrupting ROS levels, which disrupted mitochondrial function and resulted in cells apoptosis in B16-F10 cells. In addition, the expression of ATP was down-regulated and the expression of apoptotic protein was up-regulated (Additional file 1: Fig. S13).

### Evaluation of in vivo hydrogen therapeutic efficacy

**Fig. 4:**
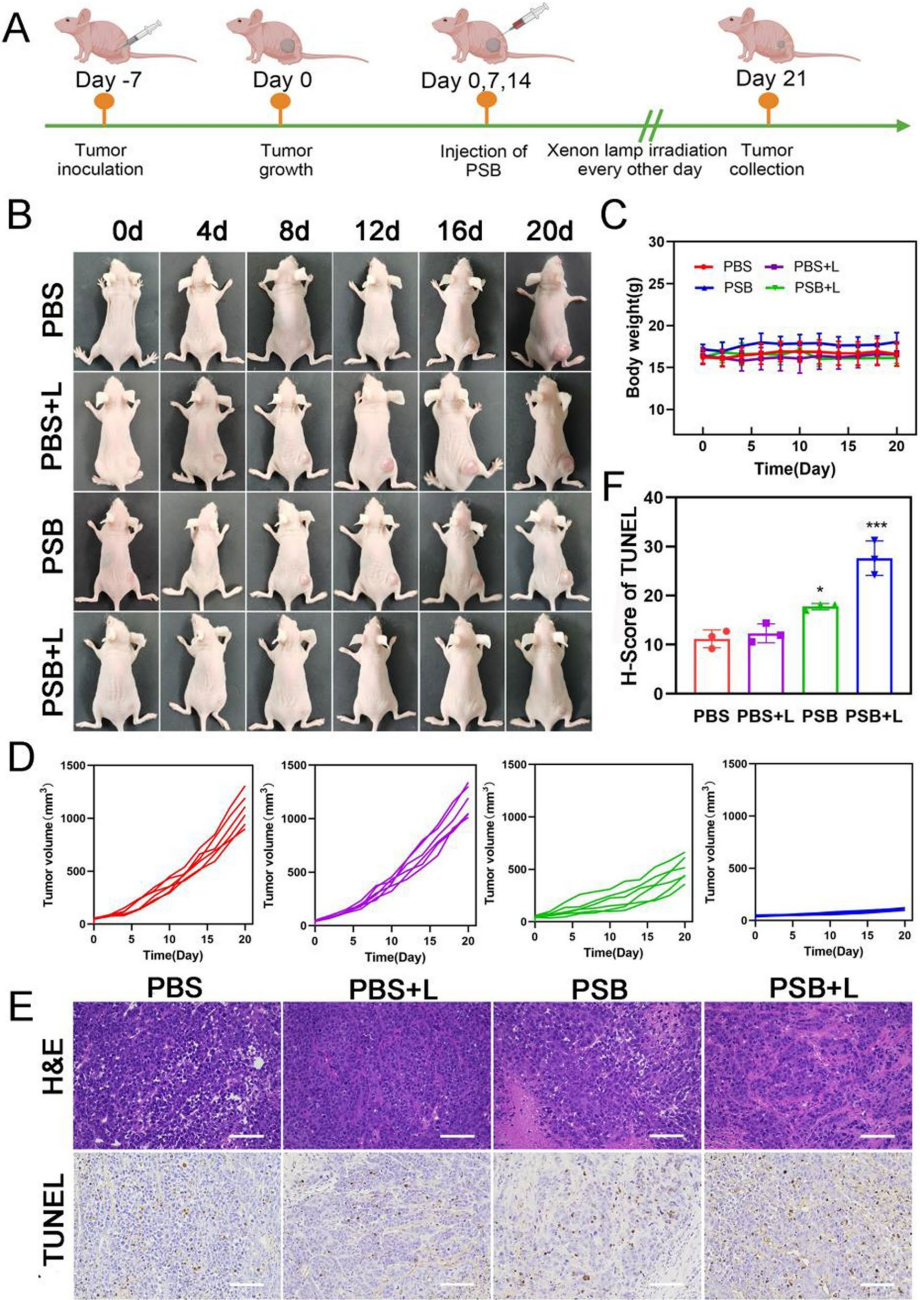
In vivo antitumor study. **A** Schematic diagram of MCF-7 tumor model. **B** Images of MCF-7 tumor-bearing mice in different treatment groups. **C** Body weight (n = 6) and **D** Tumor volume of each mouse in each group (n = 6). **E** Amplifying H&E and TUNEL staining assay of the tumor from the mice in all groups after treatment for 21 days. **F** Bar graph of quantitation of TUNEL positive cells. Scale bar, 100 μm. Data are presented as the mean ± s.d. (n ≥ 3). *P < 0.05; **P < 0.01; ***P < 0.001

Fluorescence imaging was performed on mice to evaluate the biological safety of PSB (Additional file 1: Fig. S14). PSB were labeled with NHS-Rhodamine B (RhB) and injected to mice. It could be seen that the PSB was retained in the tumor site and did not spread to other organs. In addition, we verified the vivo metabolism of PSB. The results showed that no fluorescence of PSB was observed on the seventh day. This supported the PSB injection cycle (Additional file 1: Fig. S15). The blood biochemistry and blood routine were tested and confirmed PSB was non-toxic (Additional file 1: Fig. S16). 30-day body weight of mice monitoring also indicated their biological safety (Additional file 1: Fig. S17). After having demonstrated their safety, the therapeutic effect of H_2_ in vivo was then studied. The MCF-7 tumor-bearing mouse model was constructed (Fig. 4A). Tumor-bearing mice were randomly divided into PBS, PBS + L, PSB, PSB + L. Samples were injected every 7 days and the light was given every 2 days with sustaining treatment for 21 days. Tumor images (Fig. 4B) and their weights (Fig. 4C) were recorded. Body weight of mice increased steadily within 21 days. The tumor volume was recorded every 2 days. As shown in Additional file 1: Fig. S18 and Fig. 4D, the PSB + L group could significantly inhibit tumor growth. Compared with the PBS group, the tumor of the PSB group was slightly reduced. That probably because PSB would stimulate the immune response and in turn suppresses tumor growth. After 21 days, the mice were euthanized and the tumor tissues and major organs were collected for H&E staining and other histological analysis. As shown in Fig. 4E, the PSB group showed slightly damaged to tumor cells. There was more tumor cells necrosis in the PSB + L group, and little tumor cells damage in the other two groups. H&E staining was performed on the major organs (Additional file 1: Fig. S19), and it was found that the treatment of four groups did not cause damage to the major organs. Terminal deoxynucleotidyl transferase (TdT)-mediated dUTP nick-end labeling staining (TUNEL) results also shown that PSB + L caused more severe cancer cells apoptosis (Fig. 4F). Our results illustrated that H_2_ therapy had a good therapeutic effect on the inhibition of cancer cell growth. By measuring the contents of Caspase-3 and Cytochrome C (Cytc) in the tumor tissues (Additional file 1: Fig. S20), we found the damaged contents of the PSB + L group was significantly higher than that of PBS group. The results were consistent with the conclusion of previous experiments and indicated that hydrogen therapy could cause apoptosis by up-regulating apoptotic proteins such as Caspase-3.

### Hydrogen therapy increased immune cell infiltration

**Fig. 5:**
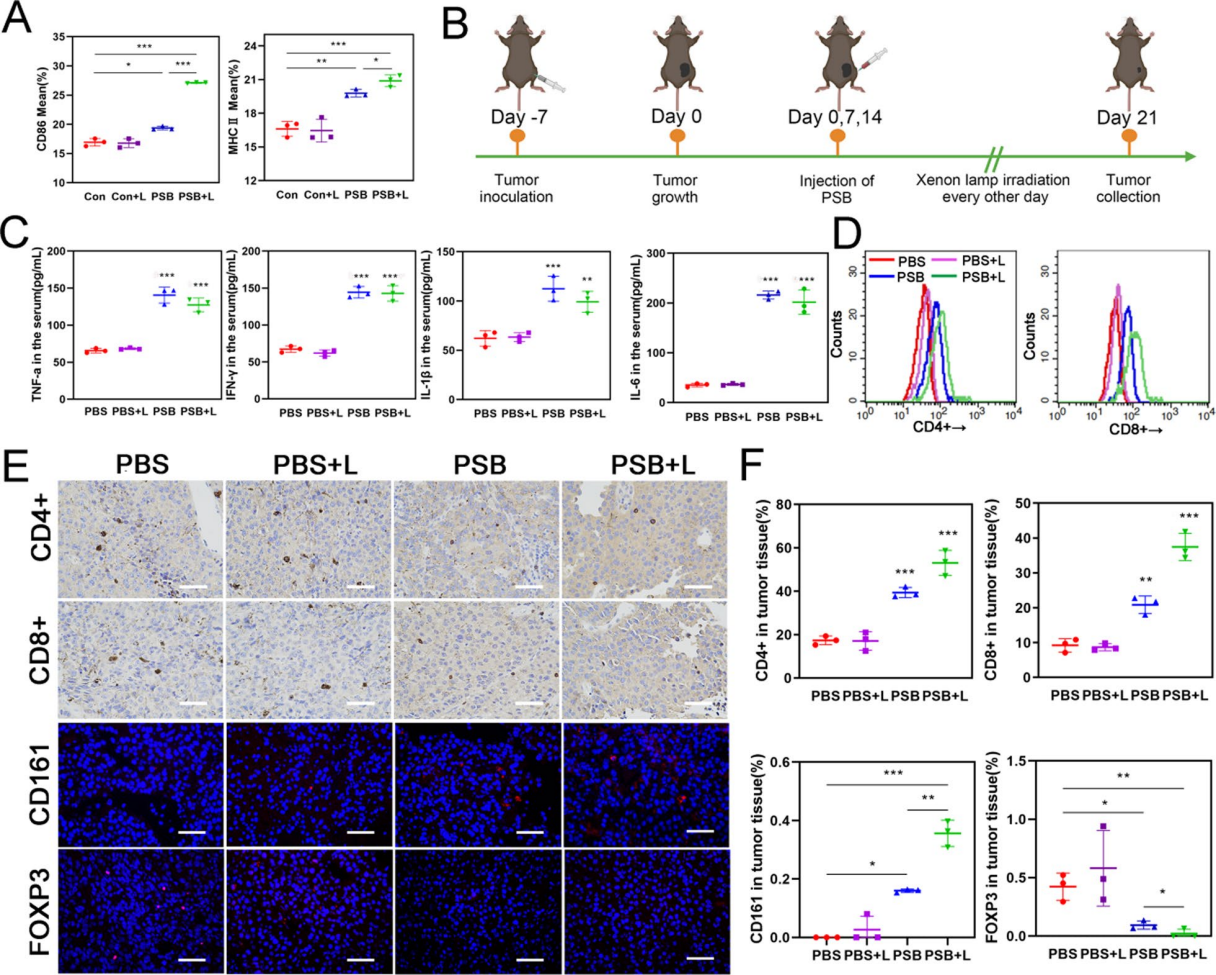
Hydrogen therapy stimulated immunity in vivo. **A** The expression of CD86 and MHC II in DC. **B** Schematic diagram of B16-F10 tumor model. **C** TNF-α; IFN-γ; IL-1β and IL-6 level in the serum. **D** Flow cytometry analysis of CD4+ T, CD8+ T cells in mice tumor tissues. **E** Immunohistochemical and immunofluorescence analysis of CD4+, CD8+, CD161 and Foxp3 in tumor tissues. Scale bar, 50 μm. **F** Bar graph of CD4+, CD8+, CD161 and Foxp3. Data are presented as the mean ± s.d. (n ≥ 3). *P < 0.05; **P < 0.01; ***P < 0.001

Firstly, we demonstrated in vitro that hydrogen therapy could promote an immune response. As shown in Fig. 5A and Additional file 1: Fig. S21, the expression of CD86 and MHC II proteins on DC was up-regulated. It indicated that hydrogen therapy could cause antigen released and promote the maturation of DC, thus enhancing the immune response. Then the B16-F10 mouse model was used to verify the immunotherapy effect of hydrogen (Fig. 5B). We recorded the images of the mice, survival curve, tumor volume, and mouse body weight over 21 days (Additional file 1: Fig. S22). After 21 days treatment, various cytokines in the blood of mice were tested (Fig. 5C). The results showed that the levels of cytokines such as TNF-α, IFN-γ, IL-1β and IL-6 were significantly up-regulated, indicating PSB promoted immunity by releasing cytokines. The cytokines level of the PSB + L group was slightly lower than the PSB group. It was mainly attributed that H_2_ had anti-inflammatory properties and could down-regulate the expression of certain anti-inflammatory cytokines. Then the mice tumor tissues were taken to analyze the expression levels of CD4+ T and CD8+ T cells by flow cytometry (Fig. 5D). Compared with the PBS group, the PSB group had more immune cell infiltration, indicating that PSB could indeed stimulate immunity. The immune cell infiltration level of the PSB + L group also increased compared with the PSB group, which was attributed to the release of antigen induced by H_2_ therapy. In addition, immunohistochemistry and immunofluorescence of tumor tissues were examined. As shown in Fig. 5E and F, CD4+, CD8+ and CD161 expression were increased. Results illustrated that the infiltration of CD4+, CD8+ T cells and NK cells was promoted after PSB-mediated hydrogen treatment in tumor tissue. However, the decreasing of Foxp3 expression indicated reduced Treg cells. In addition, gram staining of tumor tissue demonstrated the presence of PSB (Additional file 1: Fig. S23). These results suggested that PSB and hydrogen treatment could promote the immune response in tumor tissues.

### PSB reduced immune escape in B16-F10 cells

**Fig. 6:**
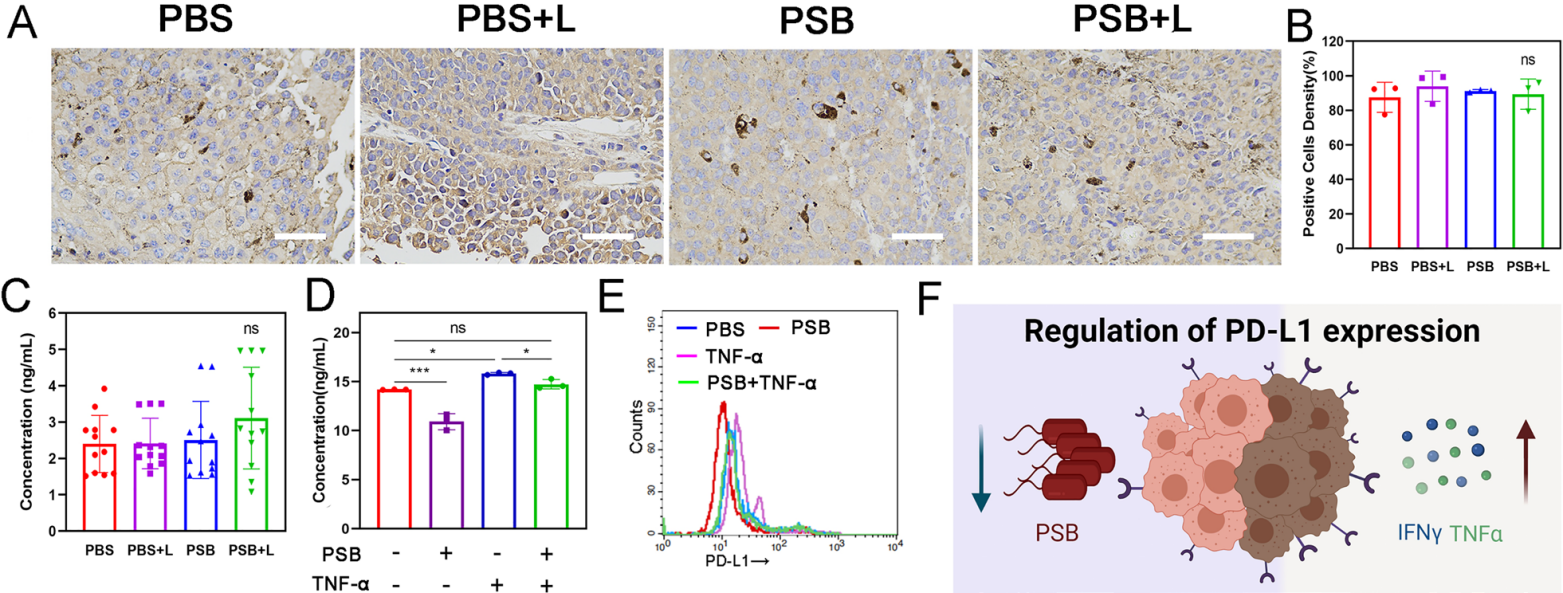
PSB-based H_2_ therapy avoided immune escape in B16-F10 mice model. **A** PD-L1 analysis in tumor tissues after different treatments. Scale bar, 50 μm. **B** Bar graph of quantitation of PD-L1 positive cells. **C** The expression levels of PD-L1 were determined by tumor tissues with PD-L1 ELISA Kit. **D** Detection of PD-L1 expression after different treatment with or without TNF-α and PSB. **E** The expression of PD-L1 was detected by flow cytometry. **F** Schematic diagram of PD-L1 expression. Data are presented as the mean ± s.d. (n ≥ 3). *P < 0.05; **P < 0.01; ***P < 0.001

The tumor tissues were taken to test the level of PD-L1 (Fig. 6A). Results showed that the PD-L1 level in the tumor tissue remained unchanged (Fig. 6B, C). Both hydrogen therapy and PSB can stimulate immunity and promote the release of TNF-α. TNF-α have been reported to up-regulate the level of PD-L1 on the surface of tumor cells. Therefore, 10 ng/mL of TNF-α and 10^7^ CFU/mL PSB were added to detect the effect on PD-L1 expression in cells. The results were found that TNF-α indeed up-regulated PD-L1, while PSB decreased the expression of PD-L1. When the PSB and TNF-α were mixed and administrated together at the same time, the expression of PD-L1 was remain unchanged comparing with the control group (Fig. 6D). Flow cytometry test also showed the same results (Fig. 6E). It was found that the expression level of PD-L1 increased with the increase of TNF-α concentration indicating a concentration-dependent manner (Additional file 1: Fig. S24). Therefore, we speculated that although cytokines such as TNF-α can up-regulate PD-L1 on the tumor cells surface, some components from PSB could down-regulate PD-L1 expression, resulting in the constant levels of PD-L1 in the final tumor tissue (Fig. 6F). This indicated that PSB could reduce the immune escape of tumor cells and have a positive effect on immunotherapy.

## Conclusions

In summary, we successfully used PSB as a microbial hydrogen production for tumor-targeted hydrogen and immune therapy. PSB could continuously release H_2_ via photosynthesis under visible light. The results showed that H_2_ had good therapeutic effects both in vitro and in vivo. More interestingly, due to the presence of PSB, PD-L1 levels will not be up-regulated, which was helpful for increasing immunotherapy and reducing immune escape. Therefore, the combination of hydrogen therapy and self-activated immunotherapy provides a new strategy for cancer treatment.

## Methods

### Measurement of H_2_ release using methylene blue (MB) probe

Under the catalysis of Pt, blue MB can be quickly reduced to colorless MBH_2_ by H_2_ [42]. According to this feature, the MB probe usually detects the H_2_ release based on the color change during the MB hydrogenation process. The MB solution had a characteristic absorption peak at 664 nm, and the decrease in absorbance was related to the release of hydrogen. 10^7^ CFU/mL of PSB was dispersed in 4 mL MB solution (9 μg/mL), and then 30 g/L glucose and 100 μg/mL Pt solution were added. The solution was irradiated with a xenon lamp at 14 A for 70 min. UV–Vis spectrum was used to detect the absorption peak at 664 nm every 10 min. The amount of hydrogen produced was detected by the decrease in the intensity of the absorption peak. The amount of hydrogen released under different light sources (808 laser and LED), different light intensities (12, 13 and 14 A) and different glucose concentrations (0, 15, and 30 g/L) were also detected using the above-mentioned method.

### H_2_ release of PSB in MCF-7 cells

MCF-7 cells were cultured in a 6-well plate, and 9 μg/mL of MB-Pt solution was added to incubate in serum-free medium for 3 h. The cell uptake of MB-Pt was stained blue under the microscope. Divide the cells into four groups: Con, Con + L, PSB, and PSB + L. Then xenon lamp was used as a light source to irradiate the cells at 14 A for 5 min. Cells were incubated for another 2 h for further observing the blue color under microscopy.

### Detection of intracellular ROS level

DCFH-DA fluorescent probe was used for detection of intracellular ROS. MCF-7 cells with a concentration of 1 × 10^5^ were plated in a 12-well plate until they adhered, and the changes of ROS after hydrogen treatment for different periods of time were detected. 10^7^ CFU/mL of PSB was added at 0, 1, 3, 6, and 8 h and irradiated under xenon lamp at a intensity of 14 A for 5 min. Finally, fluorescent microscopy was used to observe the fluorescence in the cells treated at different times (Nanjing Jiancheng, E004-1-1).

### Mitochondrial membrane potential

JC-1 staining was used to measure the loss of MMP. MCF-7 cells were cultured in a 6-well plate and incubated overnight. PSB (10^7^ CFU/mL) was added to two wells, one of which was given xenon lamp irradiation for 5 min at the intensity of 14 A and incubated for 6 h. Cells were stained with JC-1 (Beyotime, C2006) for 20 min and the excess dye was washed away with PBS. Red and green fluorescence were observed with a fluorescence microscope.

### Detection of intracellular ATP Level

Intracellular ATP was detected using an ATP assay kit (Beyotime, S0026). MCF-7 cells were cultured in a 6-well plate and divided into four groups: Con, Con + L, PSB, PSB + L. Cells were exposed under xenon lamp for 5 min at the intensity of 14 A and then ATP level was measured using a microplate reader according to the kit instructions.

### Measuring caspase-3 in cytoplasm

MCF-7 cells (1 × 10^5^ cells/mL) were seeded in the 6-well plate. After treatment with PSB and light, the cells were collected, and caspase-3 activity was measured by a caspase-3 assay kit according to manufacturer instructions (BestBio, BB-4106). The expressions of caspase-3 were detected at 405 nm using a microplate reader.

### In vivo systemic toxicity evaluation

To evaluate the physiological effects of PSB on mice, the mice were first injected with PSB and the blood at different time periods (0, 12, 24 h) was collect for blood biochemistry and blood routine analysis. In addition, we also studied the effect of concentrations (0, 10^5^, 10^6^, 10^7^ CFU/mL) on mice. Weight changes were recorded for 30 days.

### In vivo FL imaging

The IVIS system was used to observe the PSB fluorescence of mice in vivo. PSB (1 × 10^7^ CFU/mL) was labeled with the red fluorescence using NHS-RhB. When the tumor volume reached 0.2 cm^3^, the mice were randomly divided into two groups, and the mice were injected with RhB-labeled PSB (10^7^ CFU/mL) with peritumoral injection. IVIS was used to observe the distribution of PSB in mice at different times. After 48 h, the mice were euthanized, and the organs were removed for further vitro imaging.

### Cytochrome C and caspase-3 level detection

Tumor tissues from different groups were lysed, the supernatant was taken for further detection. The samples were subjected to Cytochrome C (Cloud-Clone, SEA594Hu) and Caspase-3 (Jianglai, JL2027596t) detection according to the operating steps in the instructions.

### Identification of DCs activation

B16-F10 cells and DCs were divided into four groups (Con, Con + L, PSB and PSB + L) and 10^7^ CFU/mL of PSB was added into B16-F10 cells. And then B16-F10 cells were irradiated for 10 min with xenon lamp (14A) and incubation for 24 h. The PSB was removed by centrifugation and the supernatant was added to DCs for incubation for 24 h. The cell supernatant was used for TNF-α and IL-6 cytokine detection, and the cells were stained with MHC II/FITC (BioLegend Cat. No. 125508) and CD86/FITC (BioLegend Cat. No. 105005) respectively. The fluorescence expression levels were detected by flow cytometry.

### Detection of cytokine levels in blood

The mice were euthanized after treatment. Blood was taken from the eyeballs, and the supernatant was collected after centrifugation. TNF-α (Bioss, bsk12002), IL-6 (Bioss, bsk12004), IFN-γ (Bioss, bsk12001), IL-1β (Bioss, bsk12015) in the blood were detected with Elisa Kit.

### Detection of PD-L1 level in B16-F10 cells

PD-L1 levels after PSB and TNF-α treatment were measured: 1 × 10^5^ cells /mL B16-F10 cells were cultured in six-well plate, and incubated overnight, and divided into four groups. Each group was added with PBS, 1 × 10^7^ CFU/mL PSB, 10 ng/mL of TNF-α cytokines, both PSB and TNF-α (Peprotech, 315-01A). After 24 h of incubation, PD-L1 ELISA Kit was used to detect the expression level of PD-L1 (Cloud Clone, SEA788Mu).

## Supplementary Information

**Additional file 1.** **Figure S1.** The gram stain of PSB. Scale bar, 10 μm. **Figure S2.** The standard curve of MB in different concentrations. **Figure S3.** UV-Vis absorbance spectra of hydrogen production at different concentrations (10^3^, 10^4^, 10^5^, 10^6^, 10^7^ CFU/mL) of PSB. **Figure S4.** UV-Vis absorbance spectra of hydrogen production at different light intensity (12 A, 13 A, 14 A) of PSB. **Figure S5.** UV-Vis absorbance spectra of hydrogen production at different glucose concentrations (0 g/L, 15 g/L, 30 g/L) of PSB. **Figure S6.** H_2_ production under different light sources. UV-Vis absorbance spectra of hydrogen production under (A) LED and (B) 808 exciting light. (C) Quantity of hydrogen production of PSB under different light sources. **Figure S7.** The time-dependent temperature changes of PSB with different concentrations of PSB under xenon lamp.** Figure S8.** Detection of the production and diffusion of H_2_ in MCF-7 cells via MB probe under different treatments. Scale bar, 500 μm. **Figure S9.** Cytotoxic effects of PSB and H_2_. (A) Toxicity of PSB to DC at different concentrations. (B) The study of MCF-7 cells killing effect at different concentrations with or without H_2_. **Figure S10.** Calcein AM and PI staining showed confocal fluorescence images of MCTSs with or without xenon lamp irradiation for 10 min. (green: living cells; red: dead cells). Scale bar,100μm. **Figure S11.** Cell apoptosis measured by flow cytometry using Annexin V/PI staining after treatment with H_2_ for 6 h. **Figure S12.** Detection of ·OH levels in MCF-7 cells after hydrogen treatment. **Figure S13.** The mechanistic of H_2_ therapy on B16-F10 cells. (A) Detection of MMP changed with JC-1 staining in B16-F10 cells. Scale bar, 400 μm. (B) ROS changed in B16-F10 cells after hydrogen treatment. Scale bar, 1000 μm. (C) Fluorescence quantification of ROS. The change of (D) ·OH, (E) ATP activity and (F) Caspase-3 released in B16-F10 cells after treatment with H_2_. **Figure S14.** In vivo fluorescence imaging of mice in control and PSB group at different time after injection. **Figure S15.** The vivo metabolism of PSB at different time points. **Figure S16.** The blood biochemistry and blood routine of ICR mice after injection PSB (10^7^ CFU/mL) for different times. **Figure S17.** Weight changes in ICR mice for 30 days after the injection of PSB with different concentrations. **Figure S18.** Tumor volume in each group after different treatments (n=6). **Figure S19.** H&E staining analysis of heart, liver, spleen, lung and kidney in different treated groups. Scale bar, 50 μm. **Figure S20. **Expression levels of Caspase-3 and Cytochrome C after the different treatments in vivo. **Figure S21. **Antigen stimulated DC activation. (A) The MHC II and (B) CD86 proteins were measured by flow cytometry on the DC after H_2_ treatment. **Figure S22.** The effect of H_2_-immunotherapy in vivo. (A) Images of mice within 14 days after different treatments. (B) Survival curve, (C) Tumor volume and (D) Body weight of mice after different treatments. **Figure S23.** Gram staining in the tumor tissue. Scale bar, 50 μm. **Figure S24.** Effects of different concentrations of TNF-α on PD-L1 expression.

## Data Availability

All data analyzed during this study are included in this published article and its additional file.

## References

[1] Dole M, Wilson FR, Fife WP (1975). Science hyperbaric hydrogen therapy: a possible treatment for cancer. Science.

[2] Wan WL, Lin YJ, Chen HL, Huang CC, Shih PC, Bo YR (2017). In situ nanoreactor for photosynthesizing H_2_ gas to mitigate oxidative stress in tissue inflammation. J Am Chem Soc.

[3] Shirakawa K, Kobayashi E, Ichihara G, Kitakata H, Katsumata Y, Sugai K (2022). H_2_ inhibits the formation of neutrophil extracellular traps. JACC Basic Transl Sci.

[4] Pan WZ, Li Z, Qiu S, Dai CB, Wu SY, Zheng X (2022). Octahedral Pt-MOF with Au deposition for plasmonic effect and Schottky junction enhanced hydrogenothermal therapy of rheumatoid arthritis. Mater Today Bio.

[5] Wang SH, Liu K, Zhou Q, Xu C, Gao JB, Wang Z (2021). Hydrogen-powered microswimmers for precise and active hydrogen therapy towards acute ischemic stroke. Adv Funct Mater.

[6] Alwazeer D, Liu FF, Wu XY, LeBaron TW (2021). Combating oxidative stress and inflammation in covid-19 by molecular hydrogen therapy: mechanisms and perspectives. Oxid Med Cell Longev.

[7] He YJ, Zhang B, Chen YH, Jin QF, Wu JR, Yan F (2017). Image-guided hydrogen gas delivery for protection from myocardial ischemia-reperfusion injury via microbubbles. ACS Appl Mater Interfaces.

[8] Sun Q, Cai JM, Liu SL, Liu Y, Xu WG, Tao HY (2011). Hydrogen-rich saline provides protection against hyperoxic lung injury. J Surg Res.

[9] Lin Y, Kashi A, Sakamoto T, Suzukaw K, Kakigi A, Yamaso T (2011). Hydrogen in drinking water attenuates noise-induced hearing loss in guinea pigs. Neurosci Lett.

[10] Kubota M, Shimmura S, Kubota S, Miyashita H, Kato N, Noda K (2011). Hydrogen and *N*-acetyl-l-cysteine rescue oxidative stress-induced angiogenesis in a mouse cornealalkali-burn model. Invest Ophthalmol Vis Sci.

[11] Chen CW, Chen QB, Mao YF, Xu SM, Xia CY, Shi XY (2010). Hydrogen-rich saline protects against spinal cord injury in rats. Neurochem Res.

[12] Ohsawa O, Ishikawa M, Takahashi K, Watanabe M, Nishimaki K, Yamagata K (2007). Hydrogen acts as a therapeutic antioxidant by selectively reducing cytotoxic oxygen radicals. Nat Med.

[13] Lu HT, Chen W, Liu WR, Si YC, Zhao TT, Lai XL (2020). Molecular hydrogen regulates PTEN-AKT-mTOR signaling via ROS to alleviate peritoneal dialysis-related peritoneal fibrosis. FASEB J.

[14] Kim J, Kim J, Bae JS (2016). ROS homeostasis and metabolism: a critical liaison for cancer therapy. Exp Mol Med.

[15] Cui J, Chen X, Zhai X, Shi DC, Zhang RJ, Zhi X (2016). Inhalation of water electrolysis-derived hydrogen ameliorates cerebral ischemia-reperfusion injury in rats—a possible new hydrogen resource for clinical use. Neuroscience.

[16] Gao Q, Song H, Wang XT, Liang Y, Xi YJ, Gao Y (2017). Molecular hydrogen increases resilience to stress in mice. Sci Rep.

[17] Zhao PH, Jin ZK, Chen Q, Yang T, Chen DY, Meng J (2018). Local generation of hydrogen for enhanced photothermal therapy. Nat Commun.

[18] Yu SM, Li GW, Zhao PH, Cheng QK, He QJ, Ma D (2019). NIR-laser-controlled hydrogen-releasing PdH nanohydride for synergistic hydrogen-photothermal antibacterial and wound-healing therapies. Adv Funct Mater.

[19] Zhou GX, Wang YS, Jin ZK, Zhao PH, Zhang H, Wen YY (2019). Porphyrin–palladium hydride MOF nanoparticles for tumor-targeting photoacoustic imaging-guided hydrogenothermal cancer therapy. Nanoscale Horiz.

[20] Kou Z, Zhao PH, Wang ZH, Jin ZK, Chen LH, Su BL (2019). Acid-responsive H_2_-releasing Fe nanoparticles for safe and effective cancer therapy. J Mater Chem B.

[21] Fan MJ, Wen YY, Ye DE, Jin ZK, Zhao PH, Chen DY (2019). Acid-responsive H_2_-releasing 2D MgB_2_ nanosheet for therapeutic synergy and side effect attenuation of gastric cancer chemotherapy. Adv Healthc Mater.

[22] Li ZT, Wang TX, Liu J, Rawding P, Bu J, Hong S (2021). Chemically and biologically engineered bacteria-based delivery systems for emerging diagnosis and advanced therapy. Adv Mater.

[23] Qiao N, Du GS, Zhong XF, Sun X (2021). Recombinant lactic acid bacteria as promising vectors for mucosal vaccination. Exploration.

[24] Suh SB, Jo A, Traore MA, Zhan Y, Coutermarsh-Ott SL, Ringel-Scaia VM (2019). Nanoscale bacteria-enabled autonomous drug delivery system (NanoBEADS) enhances intratumoral transport of nanomedicine. Adv Sci.

[25] Igarashi K, Kawaguchi K, Kiyuna T, Miyake K, Miyake M, Singh AS (2018). Tumor-targeting salmonella typhimurium A1-R is a highly effective general therapeutic for undifferentiated soft tissue sarcoma patient-derived orthotopic xenograft nude-mouse models. Biochem Biophys Res Commun.

[26] Yi X, Zhou H, Chao Y, Xiong SS, Zhong J, Chai ZF (2020). Bacteria-triggered tumor-specific thrombosis to enable potent photothermal immunotherapy of cancer. Sci Adv.

[27] Chen WF, Wang Y, Qin M, Zhang XD, Zhang ZR, Sun X (2018). Bacteria-driven hypoxia targeting for combined biotherapy and photothermal therapy. ACS Nano.

[28] Shinnoh M, Horinaka M, Yasuda T, Yoshikawa S, Morita M, Yamada T (2013). Clostridium butyricum MIYAIRI 588 shows antitumor effects by enhancing the release of TRAIL from neutrophils through MMP-8. Int J Oncol.

[29] Park W, Cho S, Kang D, Han JH, Park JH, Lee B (2020). Tumor microenvironment targeting nano-bio emulsion for synergistic combinational X-Ray PDT with oncolytic bacteria therapy. Adv Healthc Mater.

[30] Yang H, Jiang F, Ji X, Wang L, Wang Y, Zhang L (2021). Genetically engineered bacterial protein nanoparticles for targeted cancer therapy. Int J Nanomed.

[31] Chowdhury S, Castro S, Coker C, Hinchliffe TE, Arpaia N, Danino T (2019). Programmable bacteria induce durable tumor regression and systemic antitumor immunity. Nat Med.

[32] Lu HF, Zhang GM, Wan T, Lu YF (2011). Influences of light and oxygen conditions on photosynthetic bacteria macromolecule degradation: different metabolic pathways. Bioresour Technol.

[33] Zhao EM, Liu HF, Jia YR, Xiao TS, Li JX, Zhao GQ (2022). Engineering a photosynthetic bacteria-incorporated hydrogel for infected wound healing. Acta Biomater.

[34] Zheng PL, Fan M, Liu HF, Zhang YH, Dai XY, Li H (2021). Self-propelled and near-infrared-phototaxic photosynthetic bacteria as photothermal agents for hypoxia-targeted cancer therapy. ACS Nano.

[35] Sunita Μ, Mitra CΚ (1993). Photoproduction of hydrogen by photosynthetic bacteria from sewage. J Biosci.

[36] Hallenbeck PC, Liu Y (2016). Recent advances in hydrogen production by photosynthetic bacteria. Int J Hydrogen Energy.

[37] Trchounian A (2015). Mechanisms for hydrogen production by different bacteria during mixed-acid and photo-fermentation and perspectives of hydrogen production biotechnology. Rev Biotechnol.

[38] Zhou FQ, Gao J, Xu ZA, Li TL, Gao A, Sun F (2021). Overcoming immune resistance by sequential prodrug nanovesicles for promoting chemoimmunotherapy of cancer. Nano Today.

[39] Chen SB, Li DD, Du XJ, He XY, Huang MW, Wang Y (2020). Carrier-free nanoassembly of doxorubicin prodrug and siRNA for combinationally inducing immunogenic cell death and reversing immunosuppression. Nano Today.

[40] Luo M, Wang F, Zhang H, Kenneth KW, Wu SC, Chen Z (2020). Mitomycin C enhanced the efficacy of PD-L1 blockade in non-small cell lung cancer. Signal Transduct Target Ther.

[41] Tong QS, Miao WM, Huang H, Luo JQ, Liu R (2021). Tumor-penetrating nanomedicine improves the chemoimmunotherapy of pancreatic cancer. Small.

[42] Seo T, Kurokawa R, Sato B (2012). A convenient method for determining the concentration of hydrogen in water: use of methylene blue with colloidal platinum. Med Gas Res.

