# Supplementary material for: Microbial hydrogen “manufactory” for enhanced gas therapy and self-activated immunotherapy via reduced immune escape
Source: J Nanobiotechnology. 2022 Jun 15;20:280. doi: 10.1186/s12951-022-01440-7 (PMC9199139; doi:10.1186/s12951-022-01440-7)
Supplement: Supplementary file 1 — Additional file 1. Figure S1. The gram stain of PSB. Scale bar, 10 μm. Figure S2. The standard curve of MB in different concentrations. Figure S3. UV-Vis absorbance spectra of hydrogen production at different concentrations (103, 104, 105, 106, 107 CFU/mL) of PSB. Figure S4. UV-Vis absorbance spectra of hydrogen production at different light intensity (12 A, 13 A, 14 A) of PSB. Figure S5. UV-Vis absorbance spectra of hydrogen production at different glucose concentrations (0 g/L, 15 g/L, 30 g/L) of PSB. Figure S6. H2 production under different light sources. UV-Vis absorbance spectra of hydrogen production under (A) LED and (B) 808 exciting light. (C) Quantity of hydrogen production of PSB under different light sources. Figure S7. The time-dependent temperature changes of PSB with different concentrations of PSB under xenon lamp. Figure S8. Detection of the production and diffusion of H2 in MCF-7 cells via MB probe under different treatments. Scale bar, 500 μm. Figure S9. Cytotoxic effects of PSB and H2. (A) Toxicity of PSB to DC at different concentrations. (B) The study of MCF-7 cells killing effect at different concentrations with or without H2. Figure S10. Calcein AM and PI staining showed confocal fluorescence images of MCTSs with or without xenon lamp irradiation for 10 min. (green: living cells; red: dead cells). Scale bar,100μm. Figure S11. Cell apoptosis measured by flow cytometry using Annexin V/PI staining after treatment with H2 for 6 h. Figure S12. Detection of ·OH levels in MCF-7 cells after hydrogen treatment. Figure S13. The mechanistic of H2 therapy on B16-F10 cells. (A) Detection of MMP changed with JC-1 staining in B16-F10 cells. Scale bar, 400 μm. (B) ROS changed in B16-F10 cells after hydrogen treatment. Scale bar, 1000 μm. (C) Fluorescence quantification of ROS. The change of (D) ·OH, (E) ATP activity and (F) Caspase-3 released in B16-F10 cells after treatment with H2. Figure S14. In vivo fluorescence imaging of mice in control and PSB group at [file 12951_2022_1440_MOESM1_ESM.docx]

Additional file

**Microbial hydrogen “manufactory” for enhanced gas therapy and self-activated immunotherapy via reduced immune escape**

*Hongyu Yan^1,2^, Miao Fan^2^, Huifang Liu^3^, Tingshan Xiao^3^, Dandan Han^2^, Ruijun Che^2^, Wei Zhang^4^, Xiaohan Zhou^1^, June Wang^1^, Chi Zhang^4^, Xinjian Yang^2^, Jinchao Zhang^2,*^, Zhenhua Li^1,*^*

1. Dongguan Institute of Clinical Cancer Research, Affiliated Dongguan Hospital, Southern Medical University, Dongguan 523059, China.
2. College of Chemistry & Environmental Science, Key Laboratory of Medicinal Chemistry and Molecular Diagnosis of Ministry of Education, Chemical Biology Key Laboratory of Hebei Province, Hebei University, Baoding 071002, P. R. China.
3. College of Pharmaceutical Science, Key Laboratory of Pharmaceutical Quality Control of Hebei Province, Hebei University, Baoding 071002, P. R. China.
4. Department of Orthopedics, Shanghai Jiao Tong University Affiliated Sixth People's Hospital, 600 Yishan Road, Shanghai 200233, China.

* Corresponding author. E-mail: jczhang6970@163.com; [zhenhuali@hbu.edu.cn](mailto:zhenhuali@hbu.edu.cn)

**Materials and methods**

**PSB isolation**

PSB were isolated from lake bottom silt. Disperse 10 g of lake bottom silt in 50 mL of sterile water, then mix it with bacterial culture medium in a ratio of 1:5 into a conical flask, and add sterilized liquid paraffin last time. the triangular flask was placed in bacterial incubator for 15-30 days at 30°C. The culture medium was transferred to the freshly prepared medium for the second enrichment culture. The final culture was isolated by streaking to obtain pure photosynthetic bacteria.

**PSB culture**

PSB was obtained from our laboratory. The PSB were Rhodobacter johrii, a species of phototrophic purple nonsulfur bacteria [34]. PSB was cultured in a sterile incubator at 37°C with paraffin oil for 7-12 days under 1000 Luke light. The LB medium contains 1 g of dipotassium hydrogen phosphate, 0.5 g of MgSO_4_·7H_2_O, and 10.0 g of yeast extract, which are dissolved in 1000 mL of tertiary water. Then, the pH of the solution was adjusted to 7.0-7.2 with 1 M sodium hydroxide, and the solution was sterilized in a sterilizing pot at 120°C for 15 minutes.

**Photothermal effect of different concentrations of PSB**

PSB with different concentrations (10^4^, 10^5^, 10^6^, 10^7^ CFU/mL) was centrifuged and washed for three times with PBS. Then PSB solutions with different concentrations was irradiated with xenon lamp and radiation intensity was 14 A. The light of xenon lamp was full of wavelength. The temperature change was monitored using a photothermal image in every 1 min. Temperature changes of the PSB solution within 8 min were recorded.

**Gram stain**

Gram-negative bacteria were stained purple by the Gram kit (BestBio). Discard the supernatant by centrifugation to collect PSB, and PSB washed with PBS for three times. The stained bacteria were observed under a microscope.

**Cell Culture**

MCF-7 cells and DC were cultured in DMEM containing 10% FBS and 1% antibiotics (penicillin-streptomycin, 10,000 U/mL) at 37°C in a humidified 5% CO_2_ atmosphere. The medium was changed every two days, and the trypsin solution was added for digestion when the cells grew to 90%.

**MCF-7 Multicellular Tumor Spheroids (MCTSs)**

MCTSs were formed according to the hanging drop culture method. When the cells reached 80% confluence, they were harvested with trypsin EDTA solution (0.25%, V/V) and centrifuged for 5 min at 800 rpm. The cells were washed three times with PBS and suspended in 12 mL of growth medium. 2 mL of the cell suspensions were transfered to ultra-low attachment 6-well plates at a concentration of 1000 cells/mL. Keep the cells grow into balls.

**The killing effect of hydrogen on MCF-7 cells**

MCF-7 cells were incubated in 96-well plates overnight at a concentration of 1×10^5^ cells/mL. They were divided into Con, Con+L, PSB and PSB+L groups. The groups of Con+L and PSB+L were irradiated with xenon lamp 14A for 5 minutes and all of groups were incubated for 6 hours. Then, the supernatant was discarded, and the PSB were washed with PBS and 10 μL of CCK-8 solution was added. After the incubation for 4 hours, the absorbance at 450 nm was measured with a microplate reader.

**Cytotoxicity measurement of PSB in Vitro**

MCF-7 cells were distributed in a 96-well plate and incubated overnight until the cells adhered to the wall. Different concentrations of PSB (10^5^, 10^6^, 10^7^, 10^8^ CFU/mL) were added to the wells respectively, and incubated for 12 hours. Then, the remaining bacteria in the wells were removed by washing 5 times with PBS, followed by sequential addition of 90 μL of fresh medium and 10 μL of MTT to each well. After dark incubation for 4 hours, the supernatant was discarded, and 100 μL of DMSO was added. The mixture was shaked at 300 rpm for 5 min and absorbance value at 570 nm was measured by the microplate reader.

**Animal model**

All animal experiments were carried out according to the institutional guidelines for animal use and approved by the institutional committee. Five-week-old female mice were purchased from Beijing River Animal Experiment Center, and three days before implantation, the cells were cultured with fresh growth medium. The cells was collected from the cell culture dish by 0.25% trypsin EDTA solution and centrifugation at 1000 rpm for 5 min. Before the experiment, the cell density was measured with a cell counter. The cells were washed three times with PBS and resuspended in physiological saline at a final concentration of 1×10^7^ cells/mL. In order to establish a tumor xenograft model, 100 μL of cell suspension was injected subcutaneously into the right side of 5-week-old BALB/c NUDE and C57BL/6J mice.

**In vivo anti-tumor study**

BALB/c NUDE mice were selected to establish a tumor-bearing model. When the tumor volume reached 80 mm^3^, the mice were randomly divided into four groups. At the beginning of the experiments, the average tumor sizes of all groups were similar (no statistical difference). The injection site was next to the tumor. The first group of mice were injected with PBS, the second group of mice were injected with PBS and give xenon lamp with 14 A irradiation intensity, the third group of mice were injected with PSB, and the fourth group of mice was injected with PSB and give xenon lamp with 14 A irradiation intensity. The body weight and tumor volume of the mice were recorded every two days to investigate the therapeutic effect of hydrogen on tumors. In order to study whether PSB can induce a systemic immune response, C57BL/6J mice were selected to establish a tumor-bearing model. The grouping was the same as above, and 100 μL of saline was injected around the tumor as a blank control group. 100 μL of PSB suspension (1×10^8^ CFU/mL) was injected into mice adjacent to the tumor. Then all the mice were irradiated with xenon lamp of 14 A irradiation intensity every two days for 10 min. After monitoring of the growth and size of the tumor for 21 days, the tumor mice were sacrificed, and 6 mice tissues were taken. Tumor local immune cell infiltration was analyzed by immunohistochemistry method. When the animals showed signs of impaired health or the tumor volume exceeds 2 cm^3^, they would be euthanized with carbon dioxide.

**Hematoxylin and Eosin Staining and TUNEL Assays**

After the mice were euthanized, tumors and organs of different groups were taken for paraffin sections for hematoxylin and eugenol (H&E) and TUNEL staining.

**Immune cell infiltration**

After the mice were euthanized, tumors from different groups were taken for paraffin sections and stained with anti-CD4, anti-CD8 and fluorescent antibody of anti-Foxp3 and anti-CD161.

**T cell infiltration studies**

After treatment, fresh tumor tissue was collected. Fresh tumor tissue was gently ground in PBS buffer, and then adhesive tissue and indelible fragments were filtered through a filter. Cell suspension was washed with PBS. The obtained cell suspensions were stained with antibodies against CD4 and CD8 antibodies. Samples were analyzed using the FCM method.


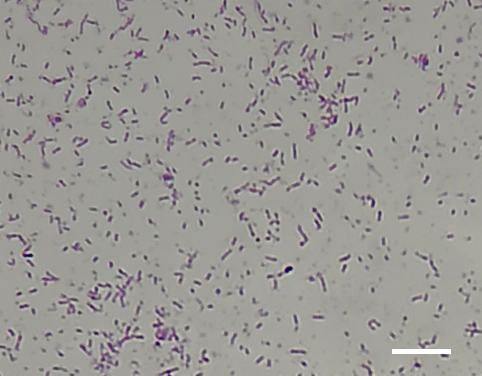


**Figure S1.** The gram stain of PSB. Scale bar, 10 μm.


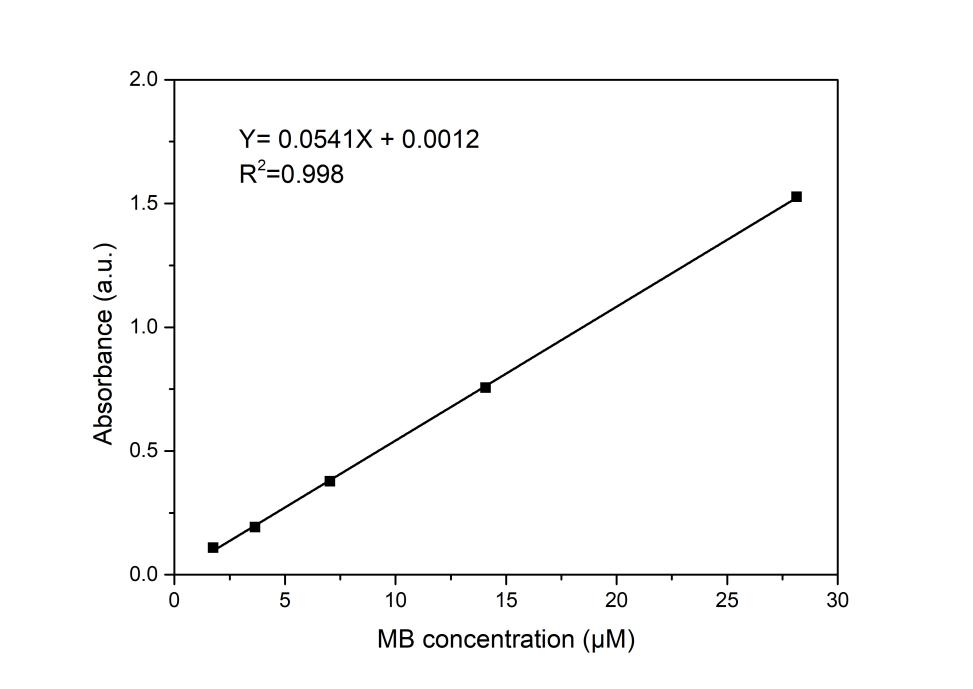


**Figure S2.** The standard curve of MB in different concentrations.


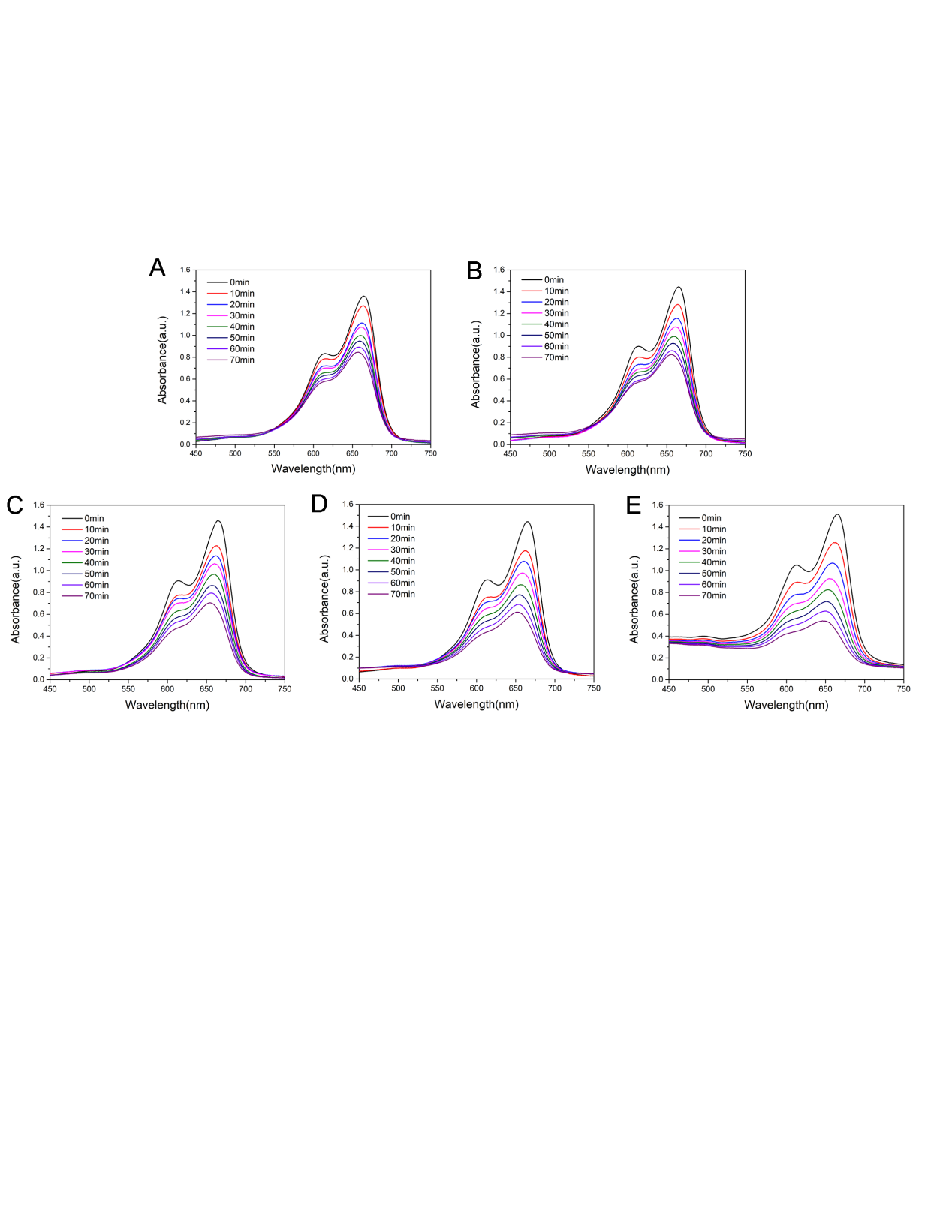


**Figure S3.** UV-Vis absorbance spectra of hydrogen production at different concentrations (10^3^, 10^4^, 10^5^, 10^6^, 10^7^ CFU/mL) of PSB.


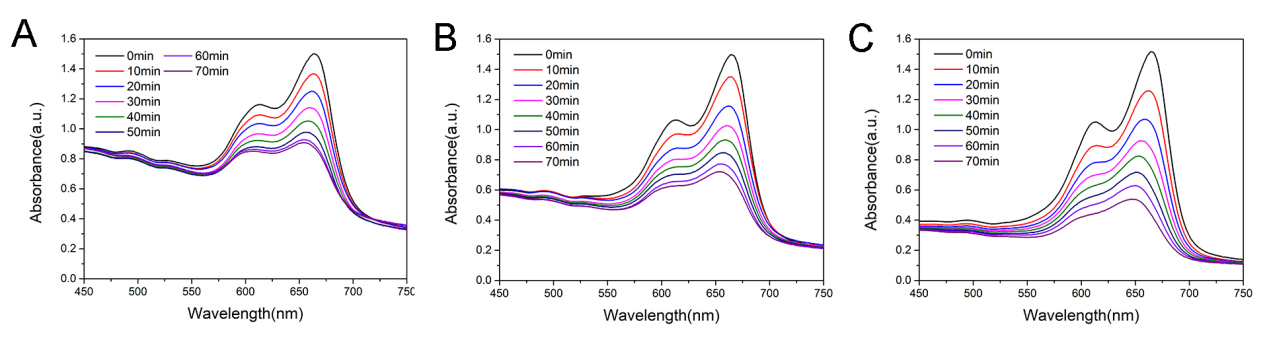


**Figure S4.** UV-Vis absorbance spectra of hydrogen production at different light intensity (12 A, 13 A, 14 A) of PSB.


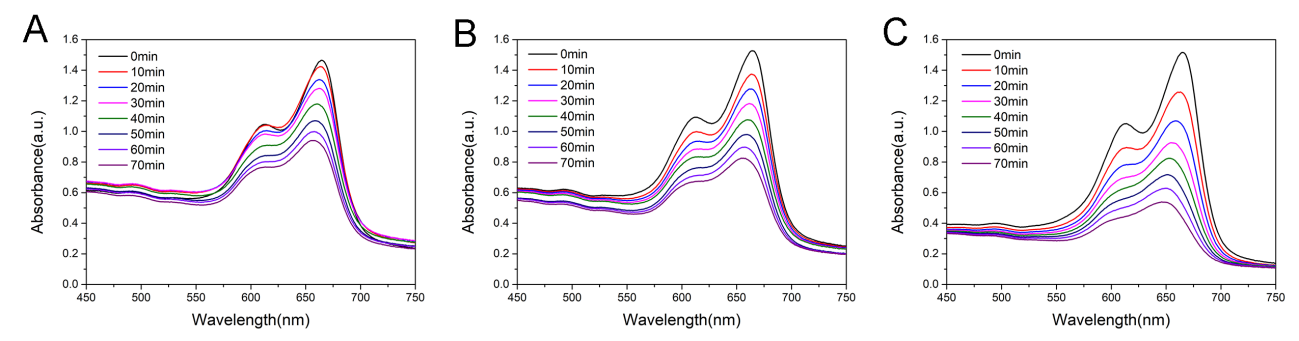


**Figure S5.** UV-Vis absorbance spectra of hydrogen production at different glucose concentrations (0 g/L, 15 g/L, 30 g/L) of PSB.


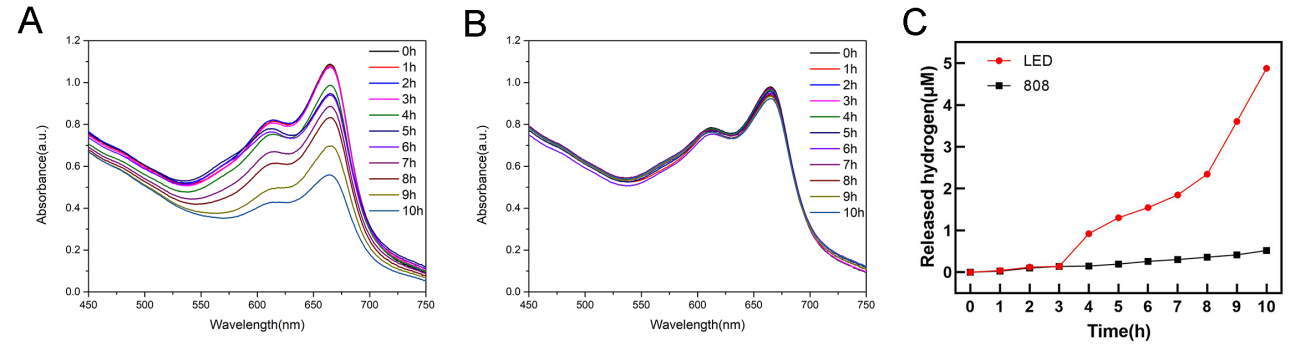


**Figure S6.** H_2_ production under different light sources. UV-Vis absorbance spectra of hydrogen production under (A) LED and (B) 808 exciting light. (C) Quantity of hydrogen production of PSB under different light sources.


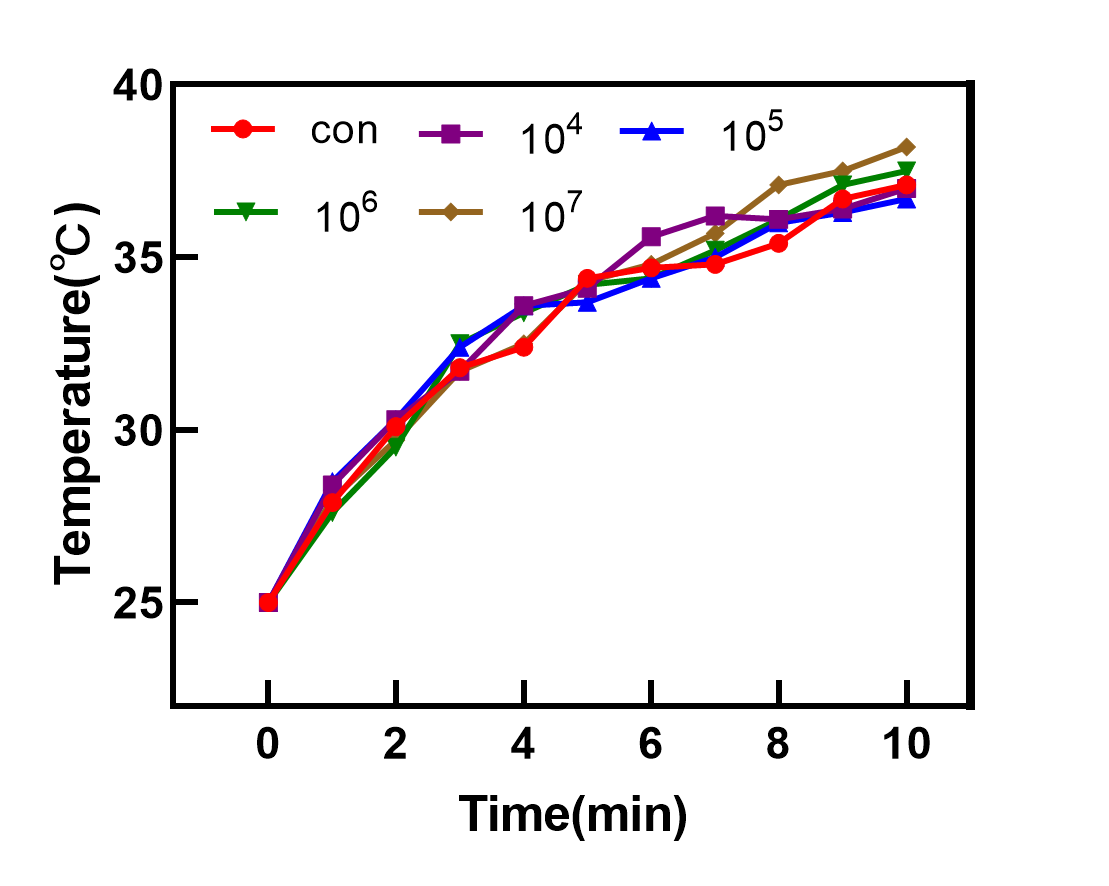


**Figure S7.** The time-dependent temperature changes of PSB with different concentrations of PSB under xenon lamp.


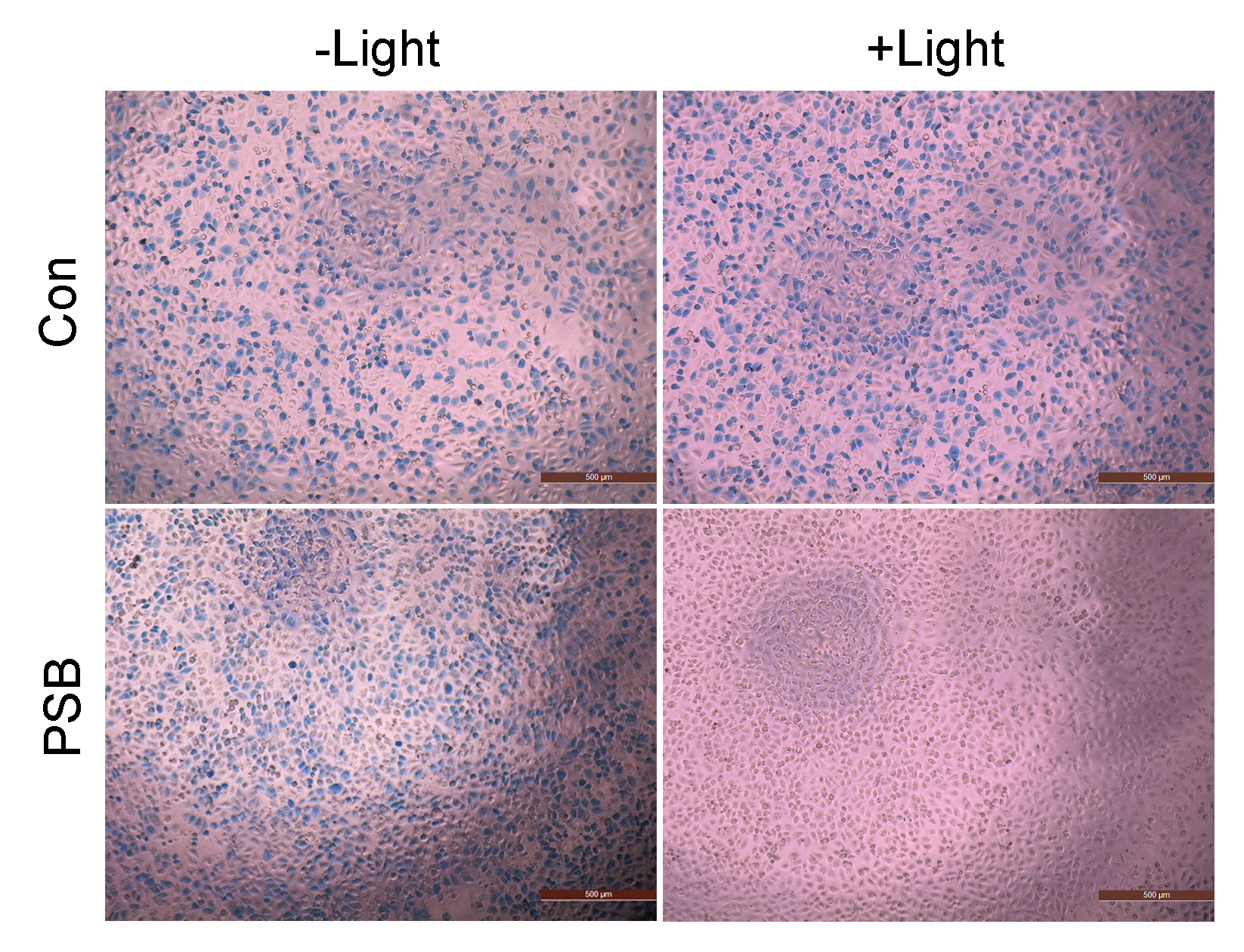


**Figure S8.** Detection of the production and diffusion of H_2_ in MCF-7 cells via MB probe under different treatments. Scale bar, 500 μm.


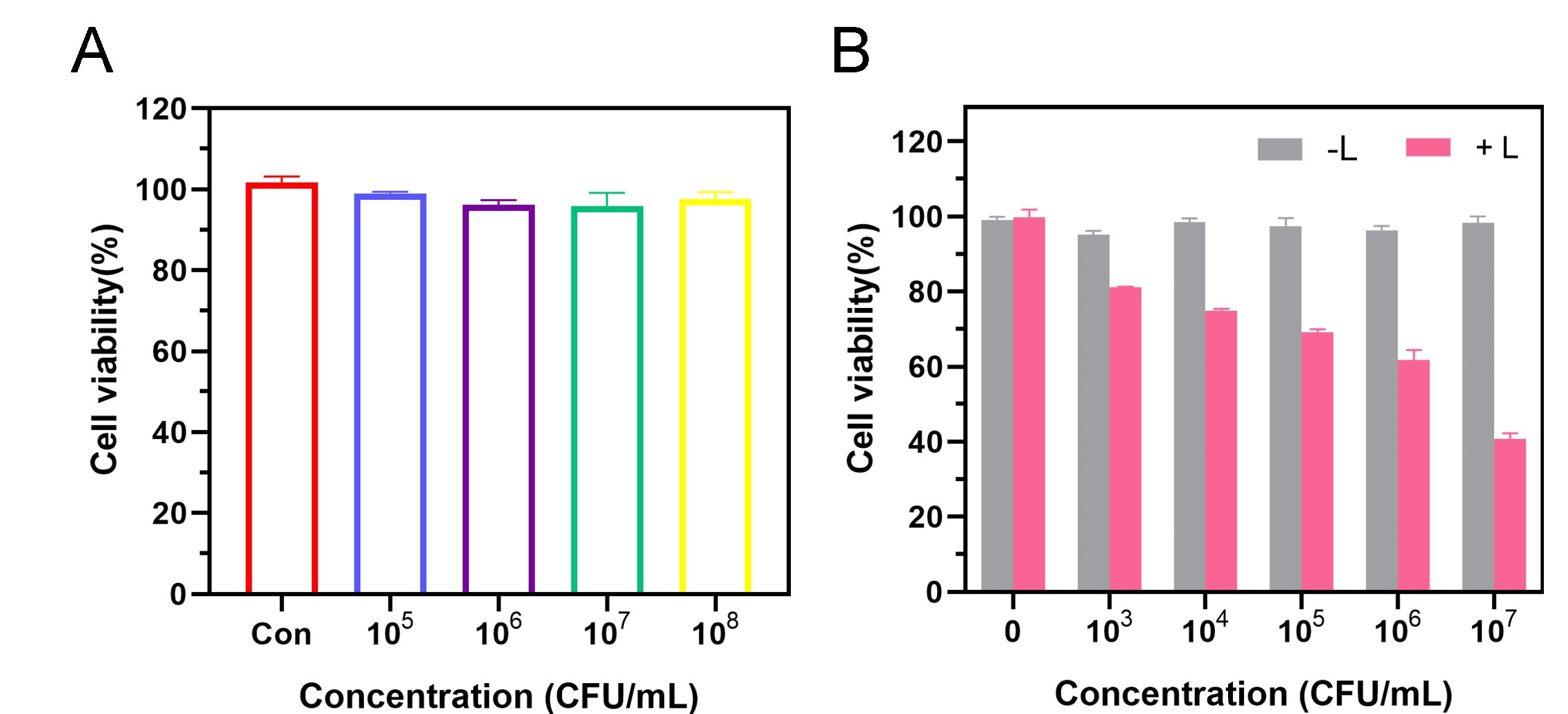


**Figure S9.** Cytotoxic effects of PSB and H_2_. (A) Toxicity of PSB to DC at different concentrations. (B) The study of MCF-7 cells killing effect at different concentrations with or without H_2_.


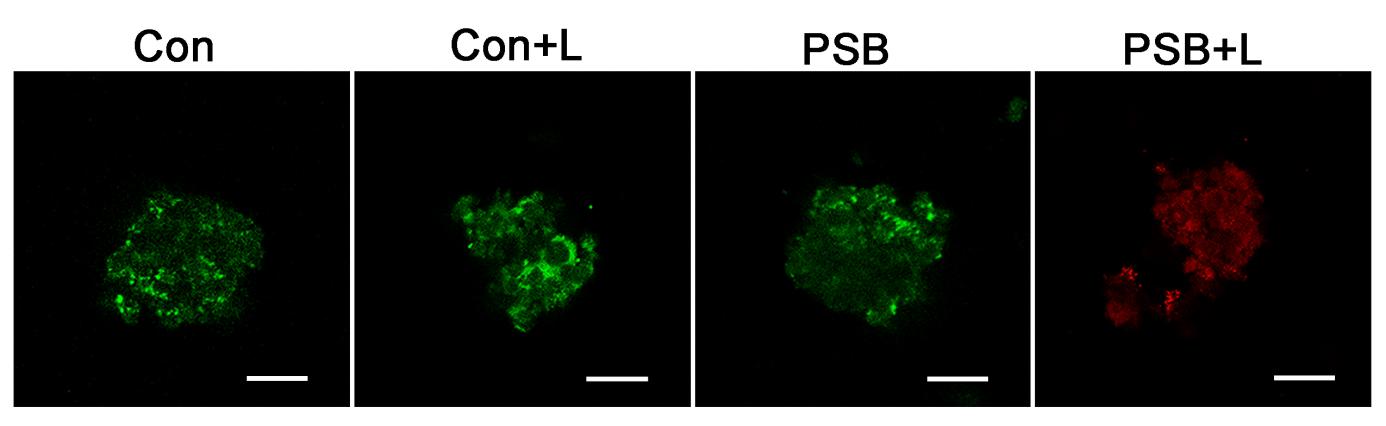


**Figure S10.** Calcein AM and PI staining showed confocal fluorescence images of MCTSs with or without xenon lamp irradiation for 10 min. (green: living cells; red: dead cells). Scale bar,100μm.


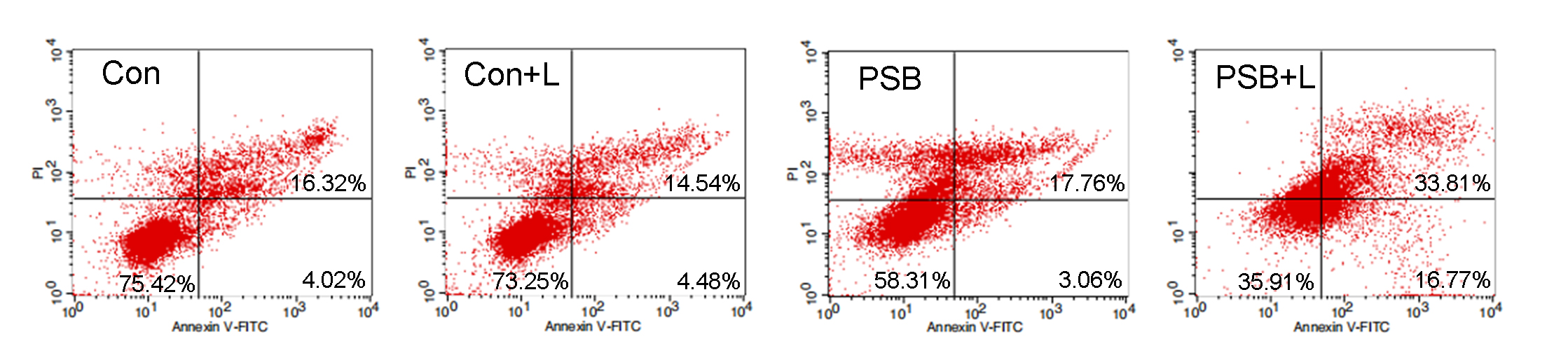


**Figure S11.** Cell apoptosis measured by flow cytometry using Annexin V/PI staining after treatment with H_2_ for 6 h.

*
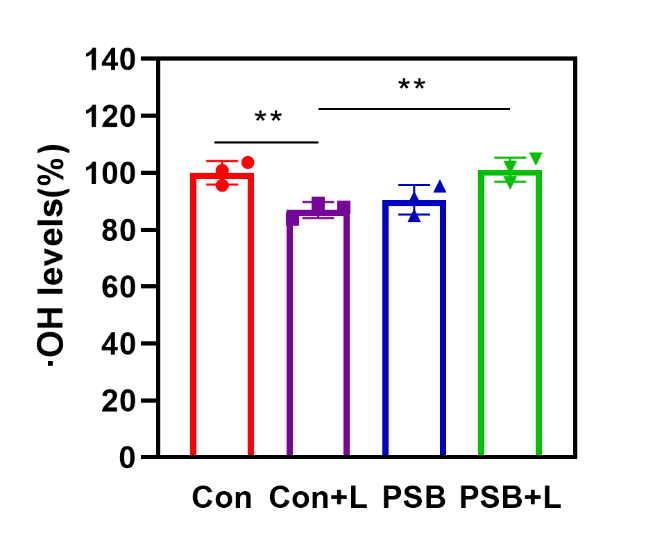
*

**Figure S12.** Detection of ·OH levels in MCF-7 cells after hydrogen treatment.


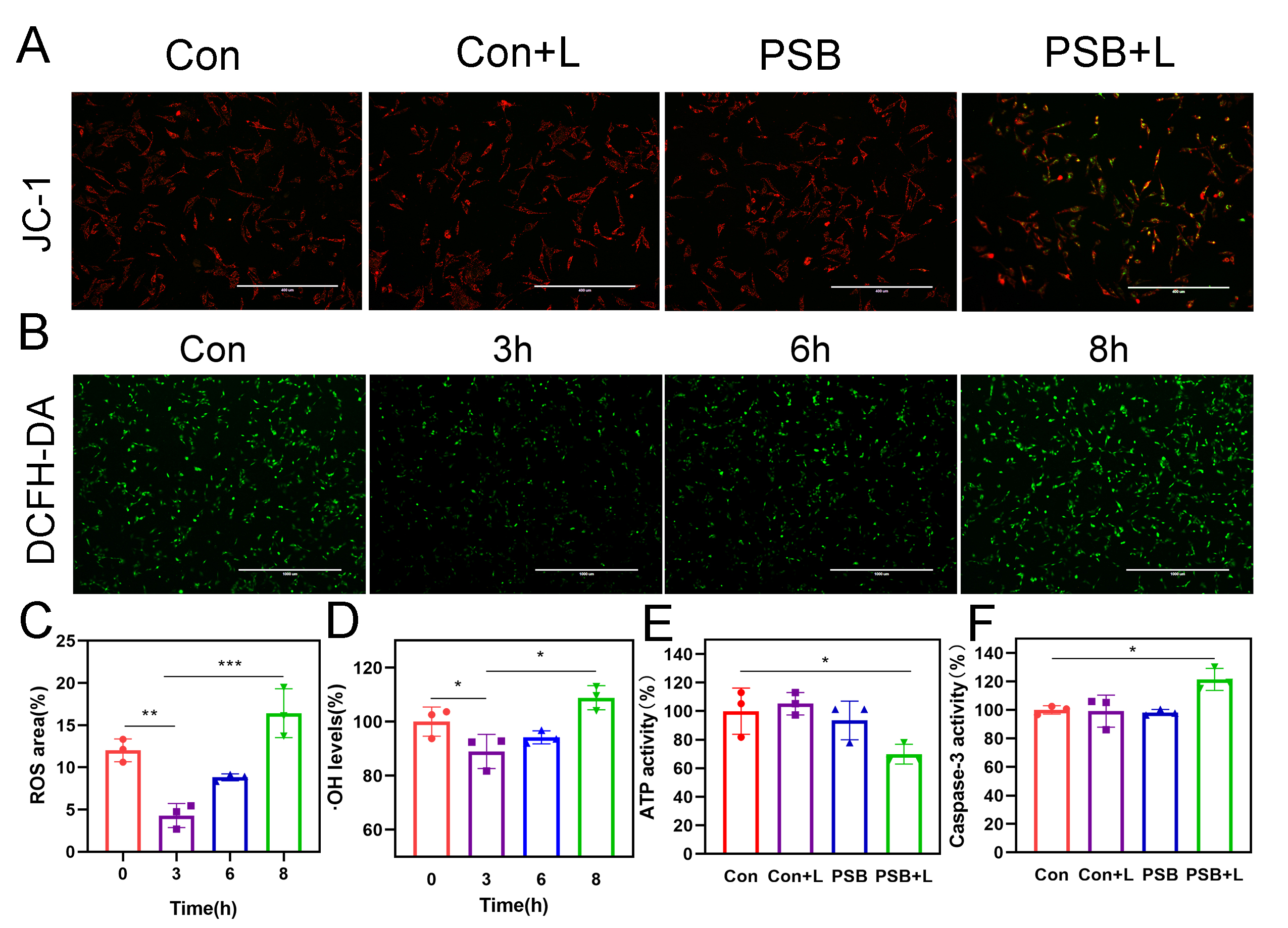


**Figure S13.** The mechanistic of H_2_ therapy on B16-F10 cells. (A) Detection of MMP changed with JC-1 staining in B16-F10 cells. Scale bar, 400 μm. (B) ROS changed in B16-F10 cells after hydrogen treatment. Scale bar, 1000 μm. (C) Fluorescence quantification of ROS. The change of (D) ·OH, (E) ATP activity and (F) Caspase-3 released in B16-F10 cells after treatment with H_2_.


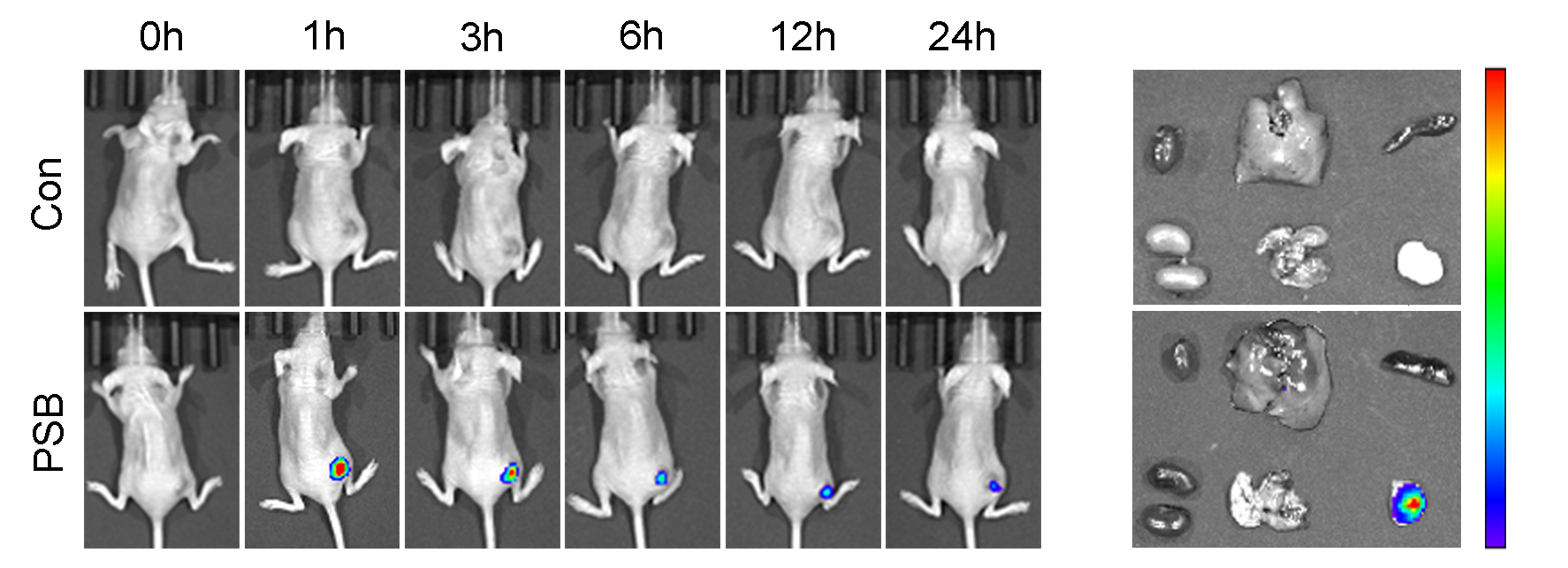


**Figure S14.** *In vivo* fluorescence imaging of mice in control and PSB group at different time after injection.


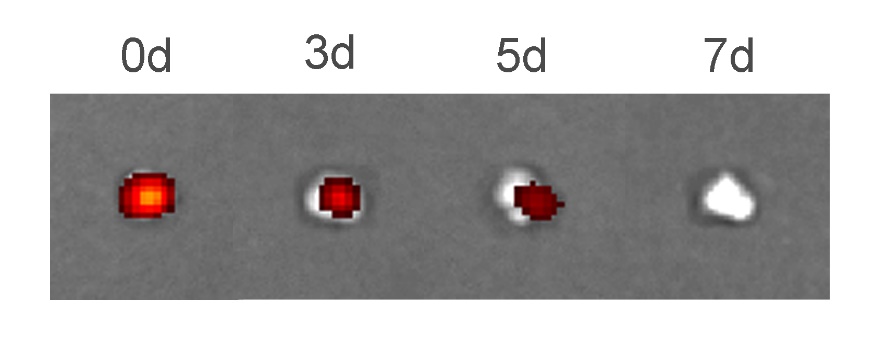


**Figure S15.** The vivo metabolism of PSB at different time points.


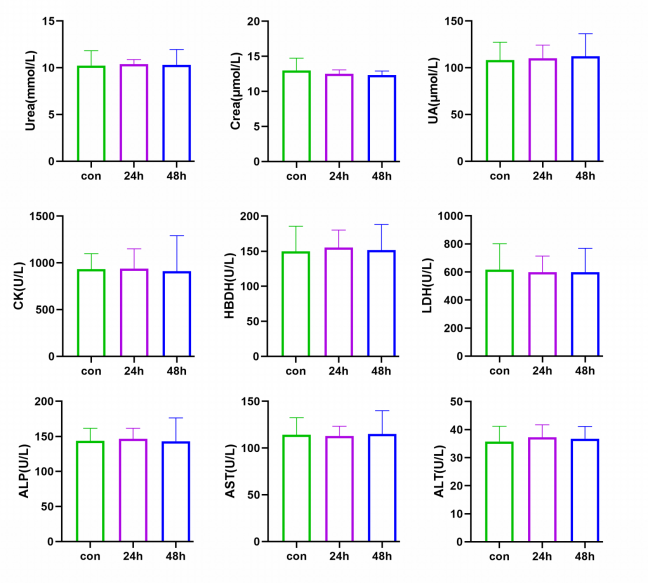

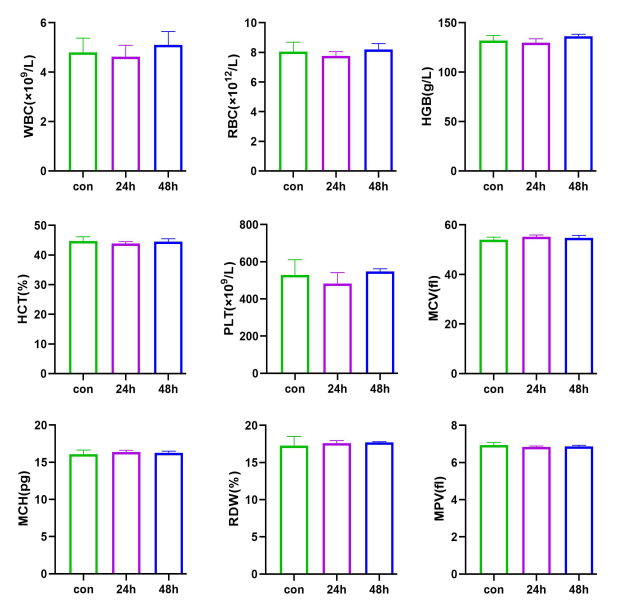


**Figure S16.** The blood biochemistry and blood routine of ICR mice after injection PSB (10^7^ CFU/mL) for different times.


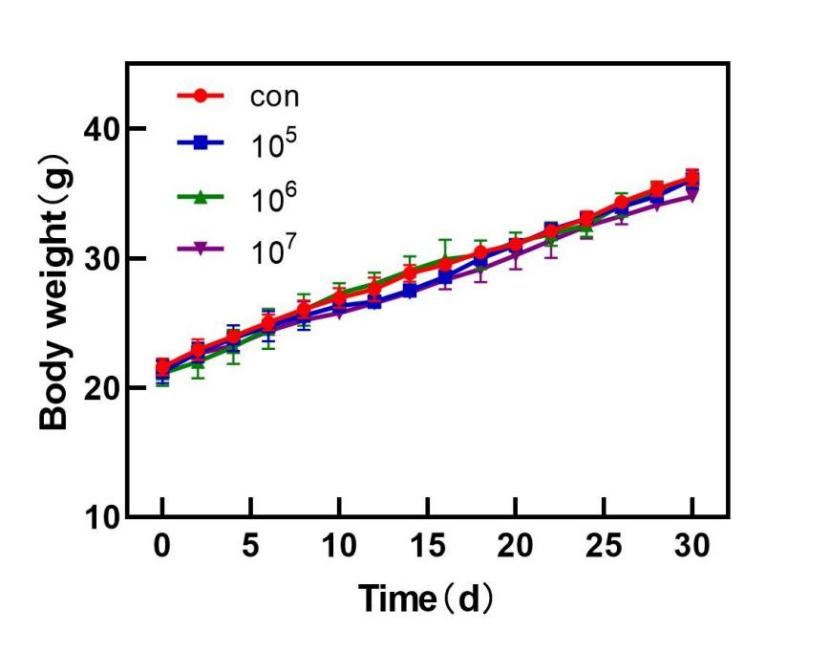


**Figure S17.** Weight changes in ICR mice for 30 days after the injection of PSB with different concentrations.


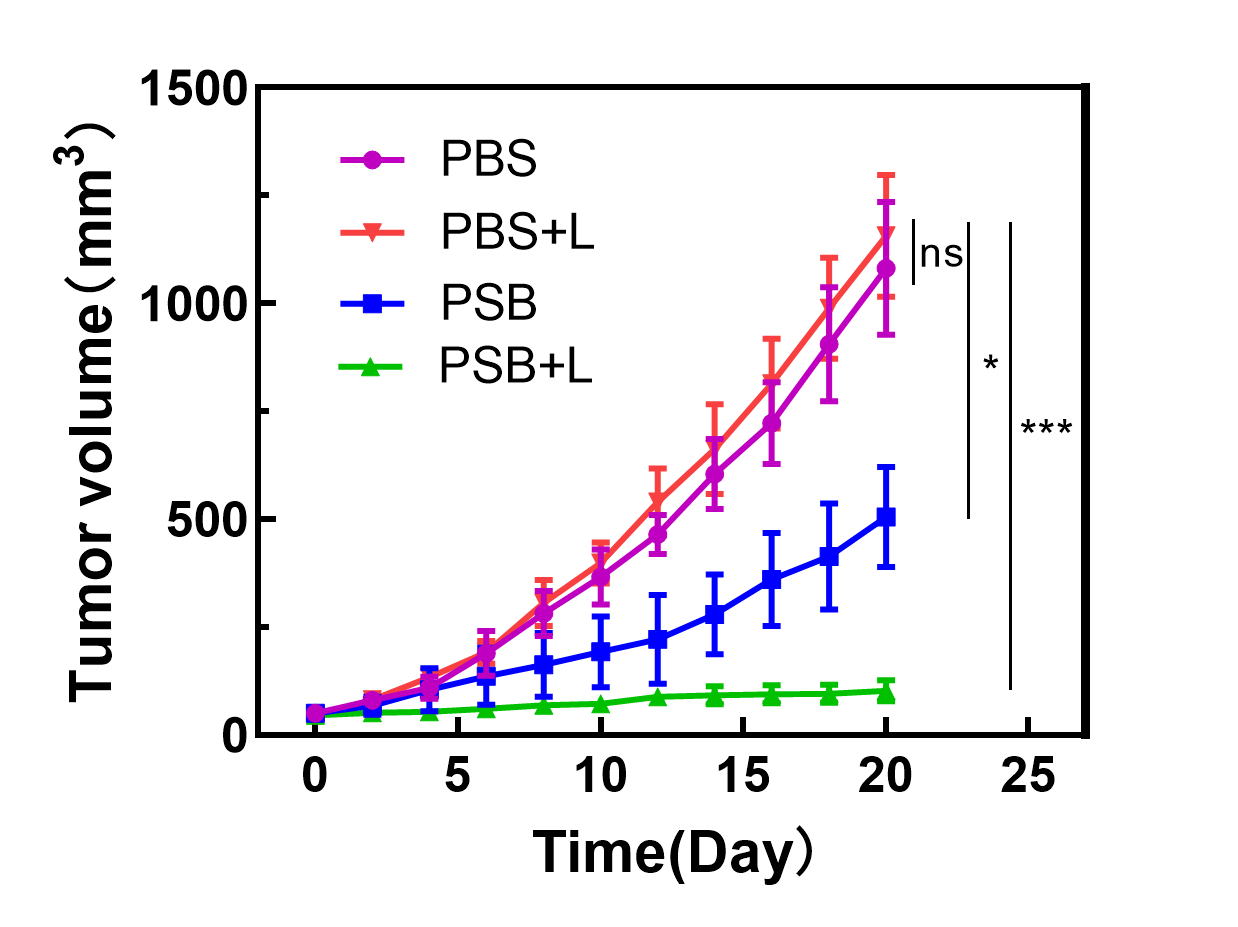


**Figure S18.** Tumor volume in each group after different treatments (n=6).


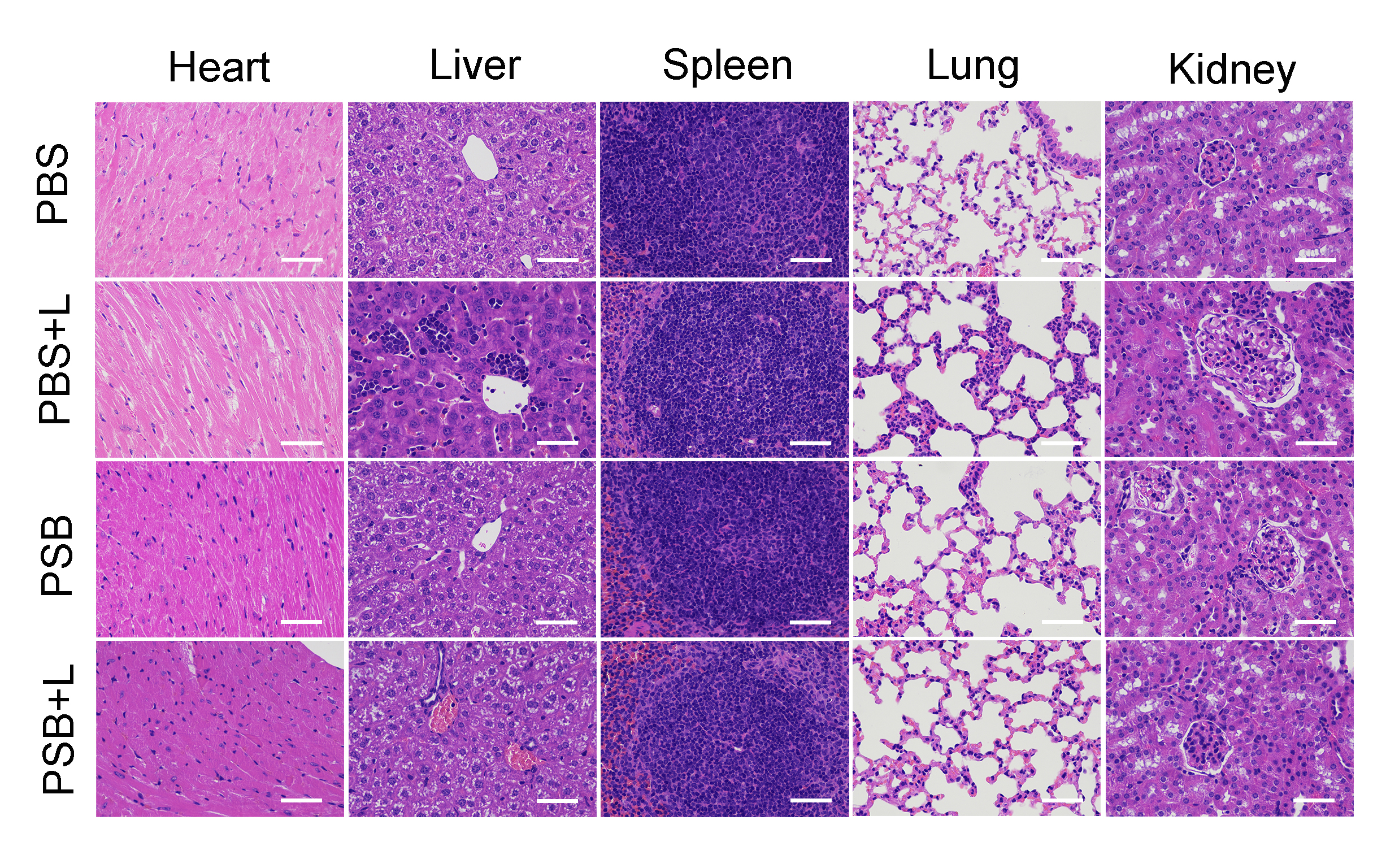


**Figure S19.** H&E staining analysis of heart, liver, spleen, lung and kidney in different treated groups. Scale bar, 50 μm.


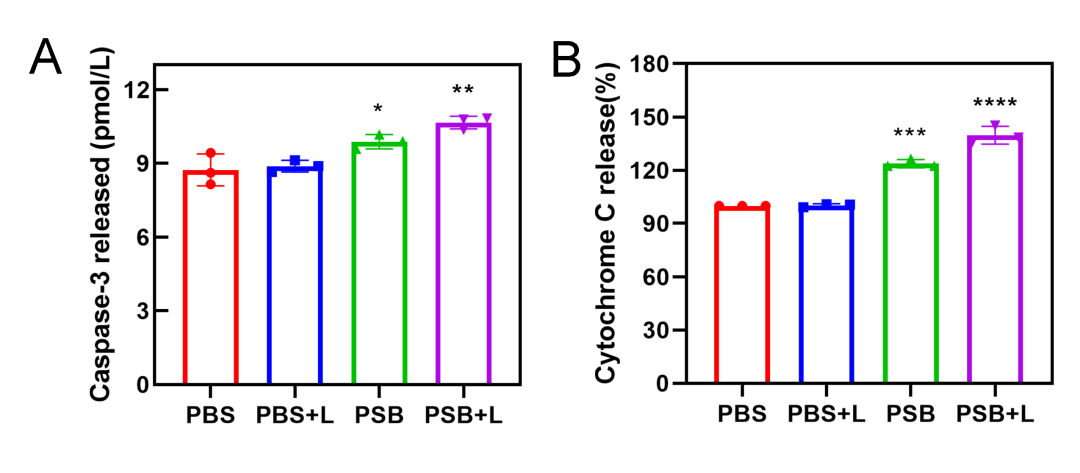


**Figure S20.** Expression levels of Caspase-3 and Cytochrome C after the different treatments *in vivo*.


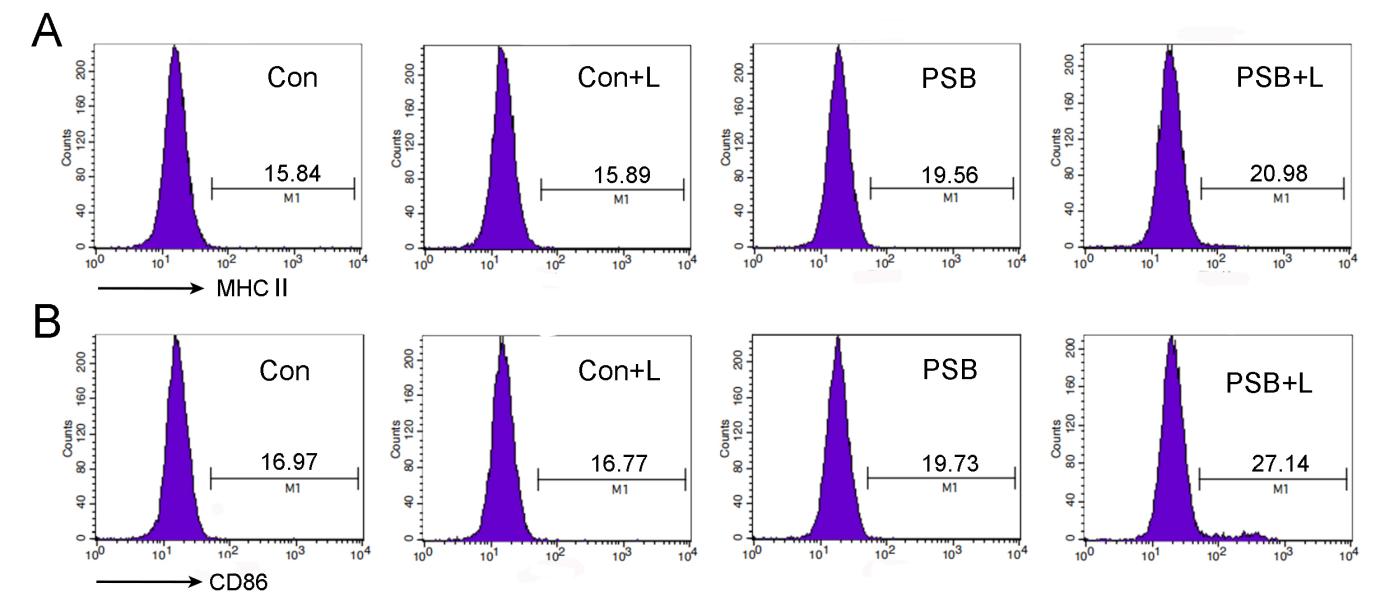


**Figure S21.** Antigen stimulated DC activation. (A) The MHC Ⅱ and (B) CD86 proteins were measured by flow cytometry on the DC after H_2_ treatment.


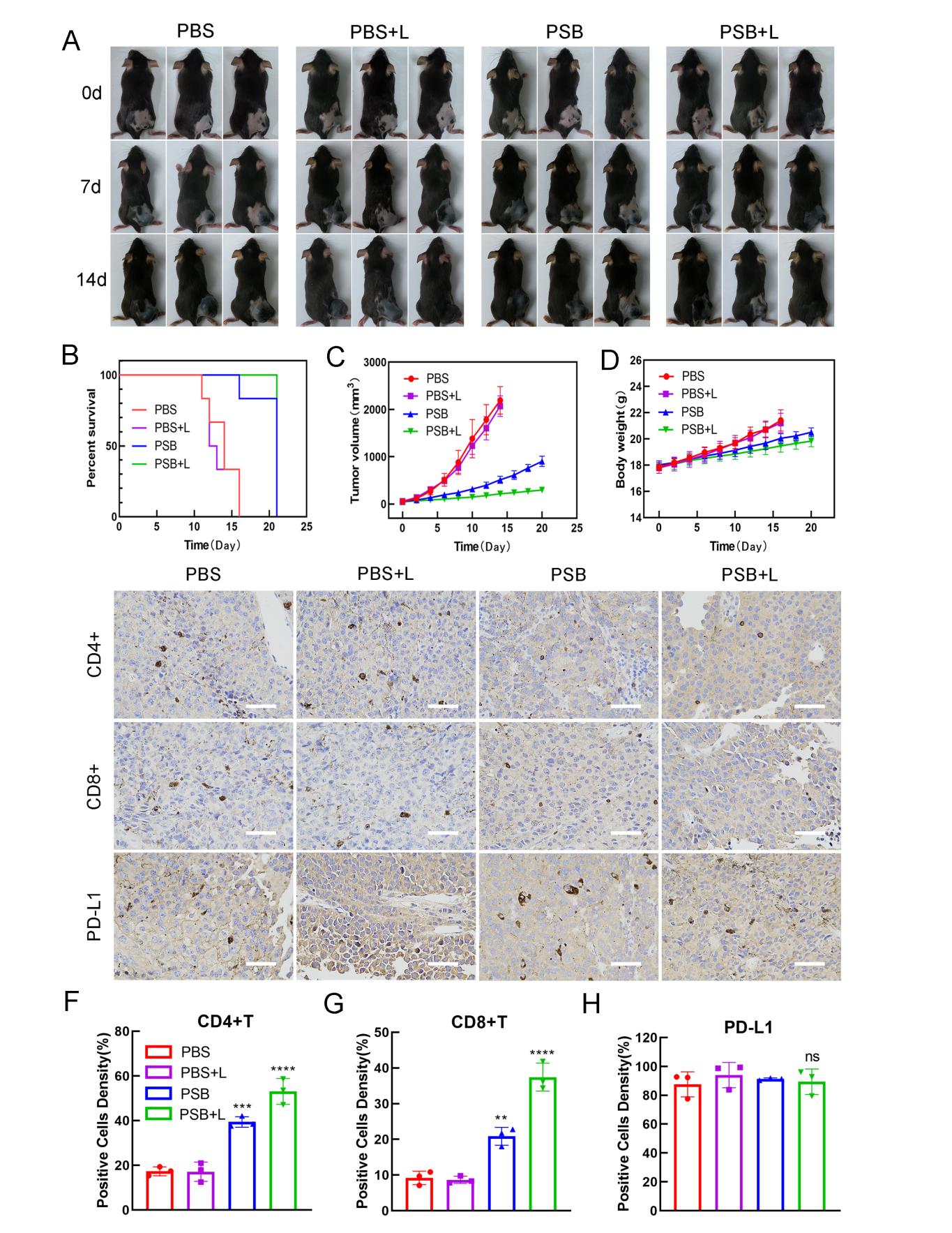


**Figure S22.** The effect of H_2_-immunotherapy *in vivo*. (A) Images of mice within 14 days after different treatments. (B) Survival curve, (C) Tumor volume and (D) Body weight of mice after different treatments.


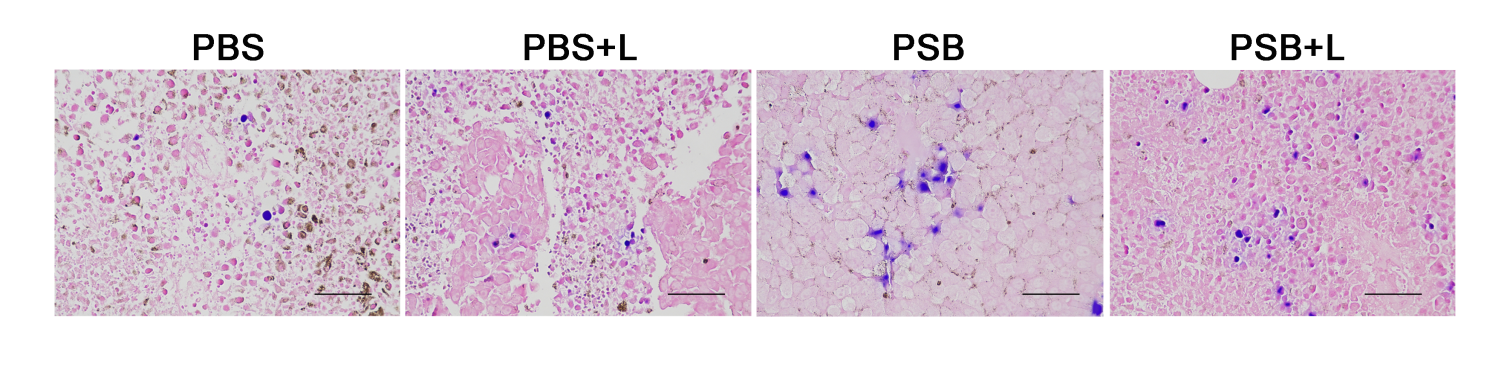


**Figure S23.** Gram staining in the tumor tissue. Scale bar, 50 μm.
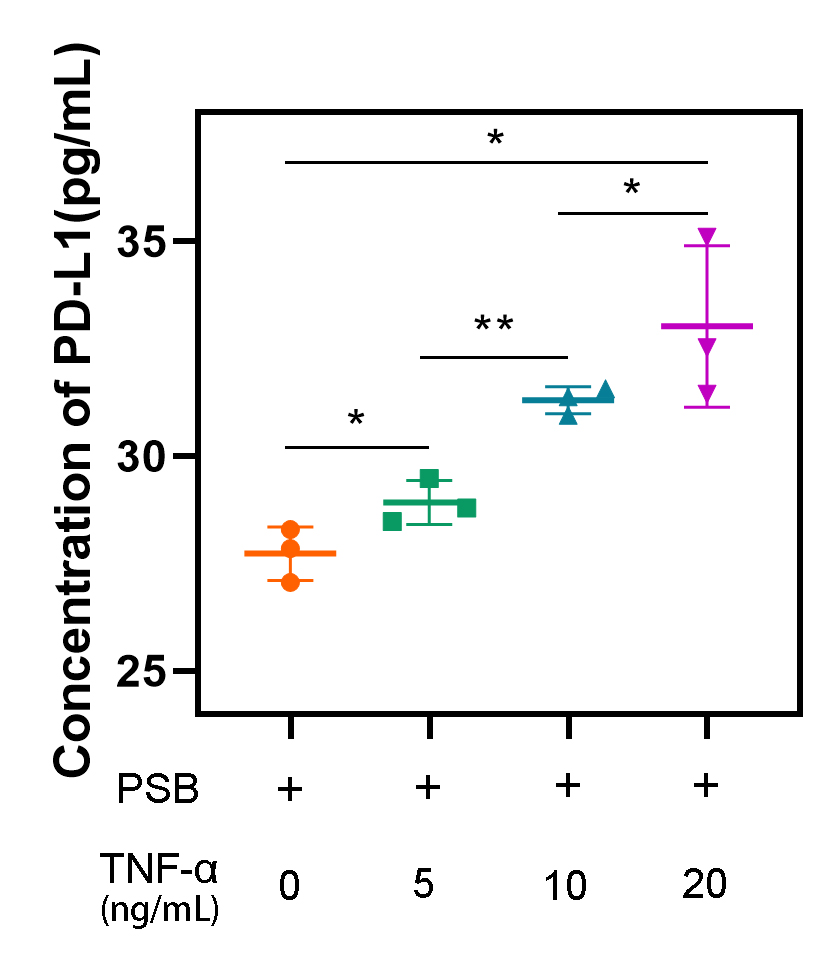


**Figure S24.** Effects of different concentrations of TNF-α on PD-L1 expression
